# Supplementary material for: Genetic inhibition of CARD9 accelerates the development of atherosclerosis in mice through CD36 dependent-defective autophagy
Source: Nat Commun. 2023 Aug 1;14:4622. doi: 10.1038/s41467-023-40216-x (PMC10394049; doi:10.1038/s41467-023-40216-x)

Supplementary figure 1

A

	% *Card9+*
Res_Macro	3.9
Inflamm. Macro	3.8
Trem2/Foamy_Mac	4.9
IFNIC	3.3
Prolif	4.1
cDC1	6.4
cDC2	7.7
Mature DCs	4.3
Monocytes	8.4
Neutro	3.3
Mast cells/Basophil	0.2
B Cells	0.0
T_Cells	1.2
Cytotoxic/NK	0.2
NK	0.0
DP_T	1.4
gdT_Cells	0.1
Fibro+ECs	0.2
RBCs	0.0
VSMC	0.0
nd	0.0

B C D

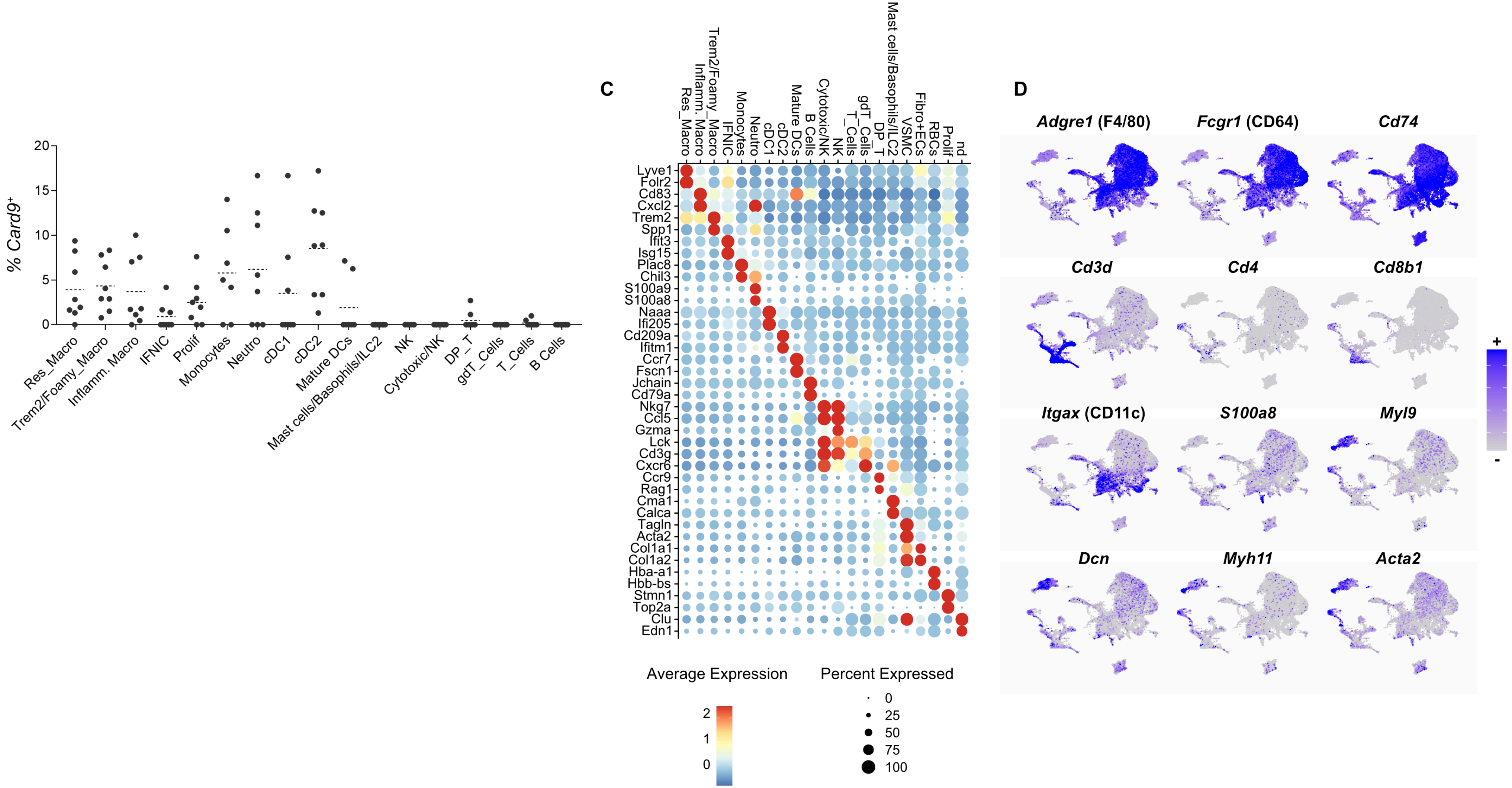

Single-cell RNA-seq analysis of *Card9* expression in mouse aortic cells in atherosclerosis. A, proportion of *Card9* expressing cells across cell types in the integrated dataset shown in **Figure 1A**; B, proportion of *Card9*+ cells across leukocyte subsets in individual datasets from **Figure 1A** were total CD45+ cells were sampled from atherosclerotic aortas (see also Zernecke et al. *Cardiovascular Research* 2022). C, Dot plot showing marker transcripts used for identification of cell identities in **Figure 1A** and **D)** expression of marker transcripts used for cell identification projected onto the UMAP plot. In D, minimum and maximum gene expression cutoffs were applied, and cells with detectable transcripts were brought to the front of plots using the "order=TRUE" argument within the Seurat FeaturePlot function.

Supplementary figure 2

A

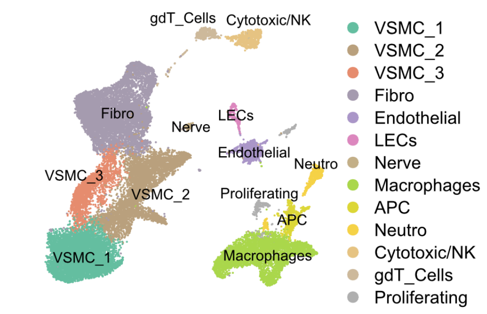

B

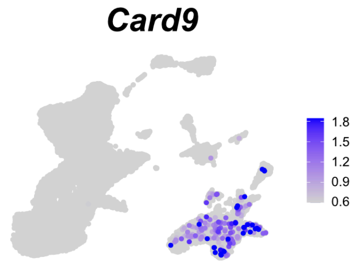

C

	% *Card9*+
APC	4.80
Cytotoxic/NK	0.00
Endothelial	0.29
Fibro	0.00
gdT_Cells	0.00
LECs	0.00
Macrophages	3.82
Nerve	0.00
Neutro	0.46
Proliferating	5.03
VSMC_1	0.00
VSMC_2	0.02
VSMC_3	0.00

D

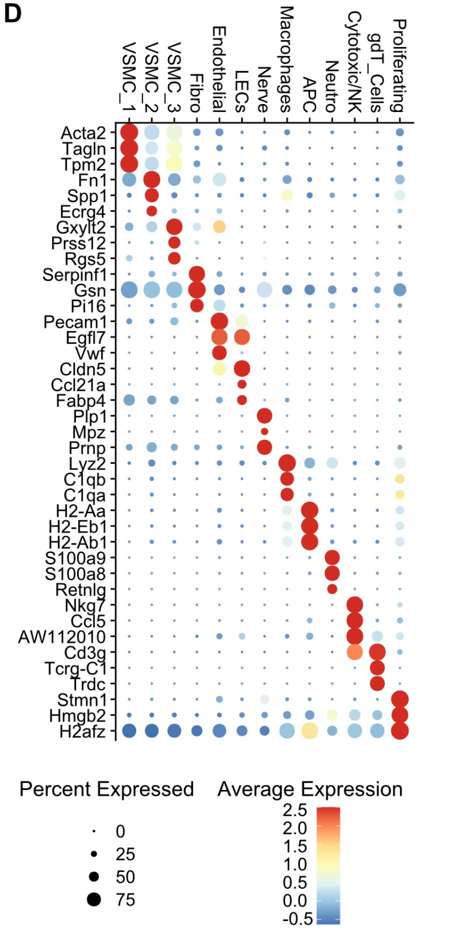

E

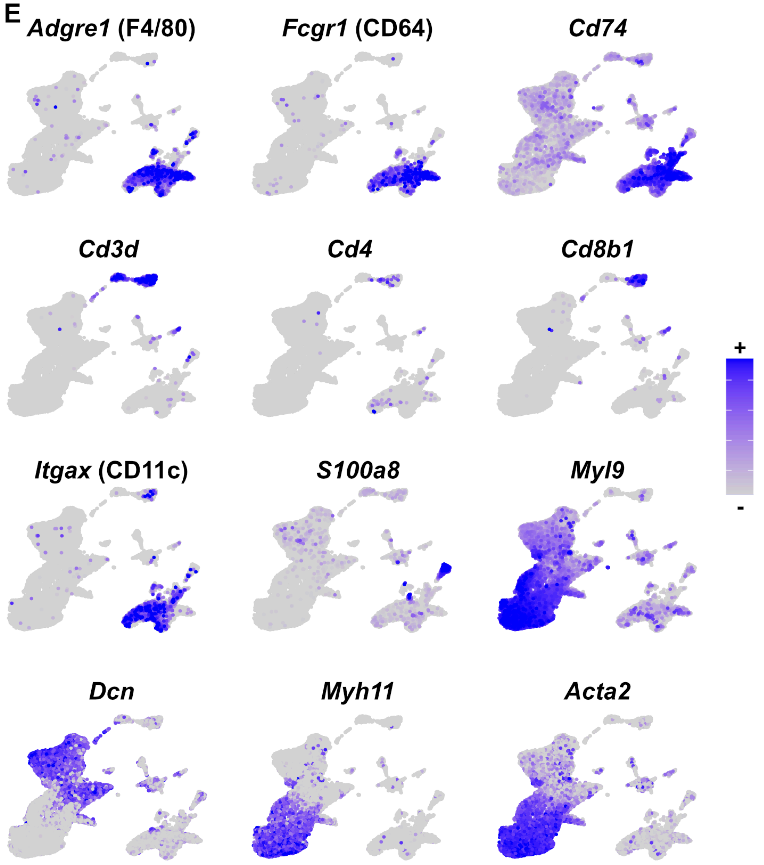

Single-cell RNA-seq analysis of *Card9* expression in mouse aortic cells in atherosclerosis in a second independent dataset. (Data from Pan et al. *Circulation* 2020). **A)** UMAP plot with cell type annotation; **B)** expression of Card9 projected onto the UMAP plot; **C)** proportion of cells with detectable *Card9* across clusters; **D)** Dot plot showing marker transcripts used for identification of cell identities and **E)** expression of marker transcripts used for cell identification projected onto the UMAP plot. In B and E, minimum and maximum gene expression cutoffs were applied, and cells with detectable transcripts were brought to the front of plots using the “order=TRUE” argument within the Seurat FeaturePlot function.

Supplementary figure 3

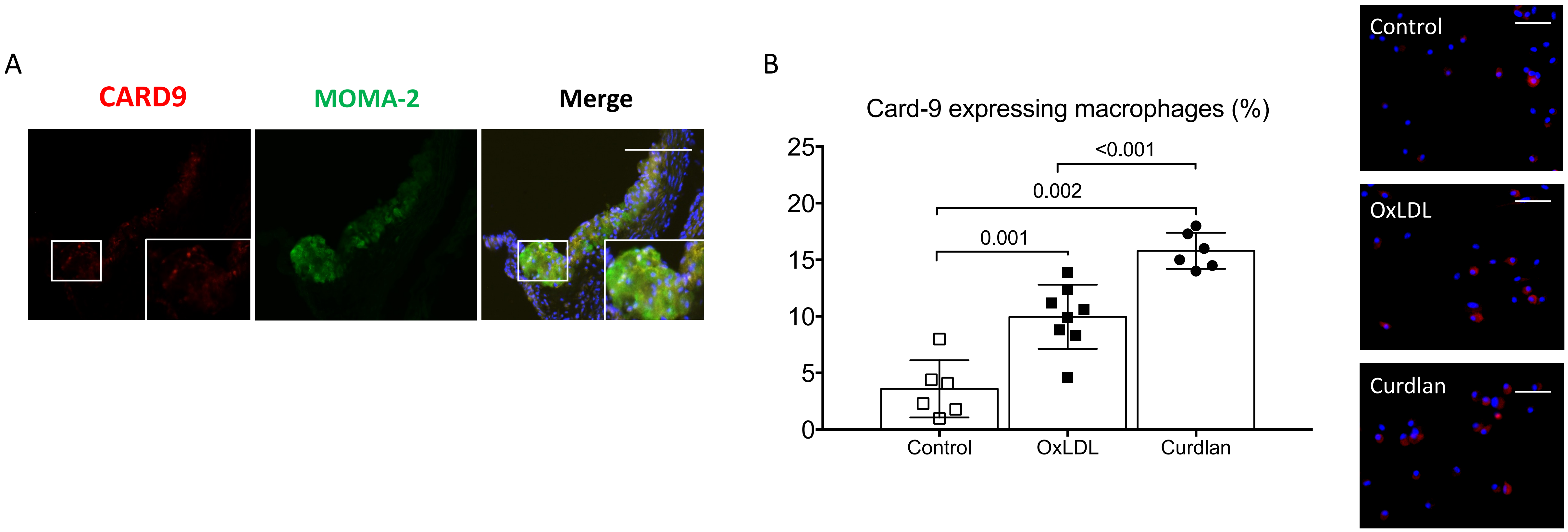

Card9 expression. A, Card9 (Red) and MOMA-2 (Green) immunofluorescent stainings in atherosclerotic plaques of 12-week old *Apoe*$^{-/-}$ mice; bar scale 50 µm (2 Pooled experiments, n=6) . B, Bone marrow-derived macrophages were stimulated in vitro during 24 hours with oxLDL (25 µg/mL) or curdlan (20 µg/mL) and Card9 expression was quantified after immunofluorescent staining (red) (n=6 Control, n=8 oxLDL and n=6 Curdlan). Bar scale 10 µm. P values were calculated using two-tailed Kruskal-Wallis test. Data are presented as mean values +/- SD. Source data are provided as a Source Data file.

Supplementary figure 4

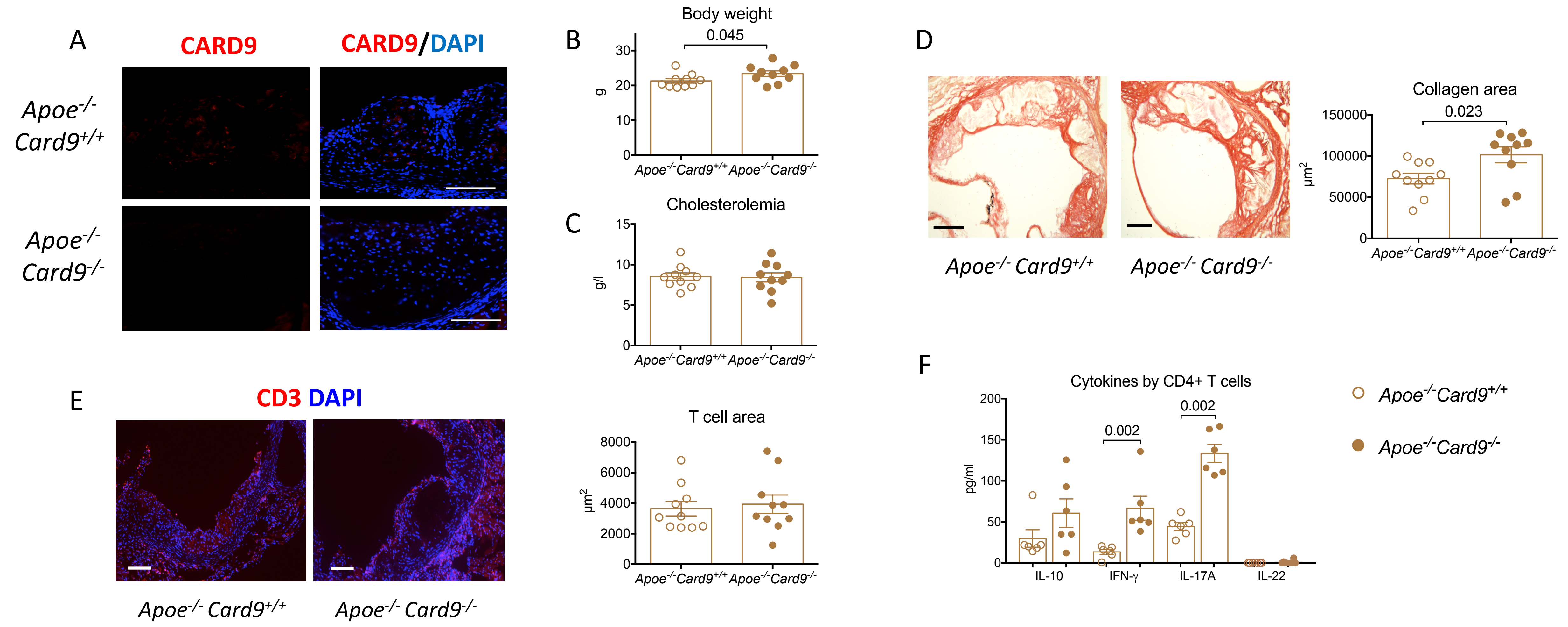

Characterization of *Apoe$^{-/-}$Card9$^{+/+}$* and *Apoe$^{-/-}$Card9$^{-/-}$* murine models. A Card9 (Red) immunofluorescent staining and DAPI (nucleus, Blue) in atherosclerotic plaques of *Apoe$^{-/-}$Card9$^{+/+}$* and *Apoe$^{-/-}$Card9$^{-/-}$* mice after 6 weeks of high fat diet; bar scale 100 µm. B and C, body weight and plasma cholesterol levels after 6 weeks of fat diet (n=10/group). D, representative photomicrographs and quantitative analysis of collagen content within atherosclerotic lesions of *Apoe$^{-/-}$ Card9$^{+/+}$* and *Apoe$^{-/-}$ Card9$^{-/-}$* mice (Sirius red staining, n=10/group). E, representative photomicrographs and quantitative analysis of CD3+ T cell infiltration (Red fluorescent staining, n=10/group). F, cytokine production (ELISA in the supernatant) of purified splenic CD4^{+} T cells from *Apoe$^{-/-}$Card9$^{+/+}$*and *Apoe$^{-/-}$Card9$^{-/-}$* animals after 48 hours of coated anti-CD3 stimulation (n=6/group). Bar scale 50 µm. Data are presented as mean values +/- SD. Two-tailed Mann-Whitney test. Source data are provided as a Source Data file.

Supplementary figure 5

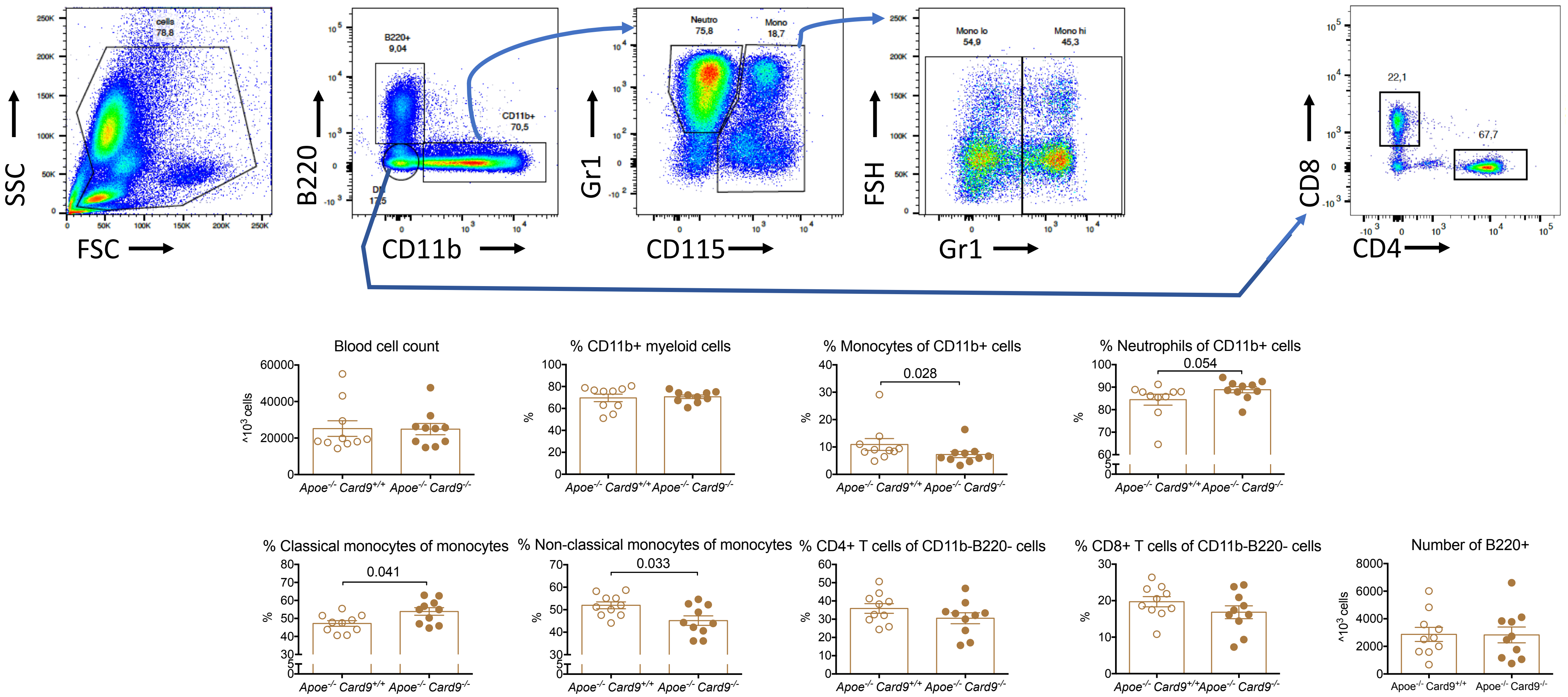

Characterization by flow cytometry of immune cell subsets in the blood of *Apoe*$^{-/-}$*Card9*$^{+/+}$ and *Apoe*$^{-/-}$*Card9*$^{-/-}$ mice. Classical monocytes were defined as CD11b+CD115+Gr1high cells; Non-classical monocytes were defined as CD11b+CD115+Gr1Low cells; neutrophils were defined as CD11b+ CD115-Gr1+ cells. CD4+ T Lymphocytes were selected as B220-CD11b-CD4+ cells, CD8+ T Lymphocytes were selected as B220-CD11b-CD8+ cells. B cells were defined as CD11b-B220+ cells. Neutrophil proportion was slightly increased in the blood of *Apoe*$^{-/-}$*Card9*$^{-/-}$ mice as well as the proportion of classical monocytes. No difference in CD4+ T, CD8+ T and B cell subsets between groups (N=10/group). Data are presented as mean values +/- SD. Two-tailed Mann-Whitney test. Source data are provided as a Source Data file.

Supplementary figure 6

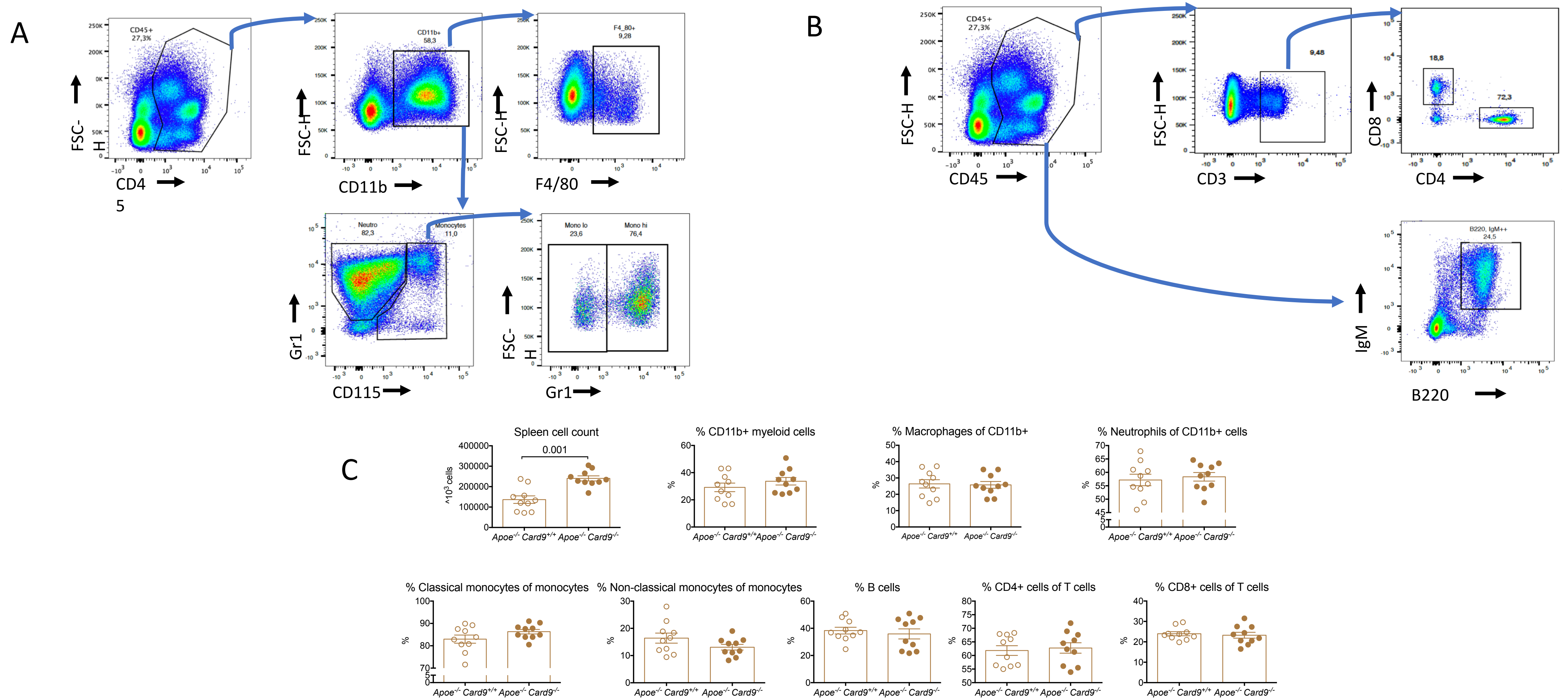

Characterization by flow cytometry of immune cell subsets in the spleen of *Apoe$^{-/-}$Card9$^{+/+}$* and *Apoe$^{-/-}$Card9$^{-/-}$* mice. A, Myeloid cells were defined as CD45+CD11b+ cells. Classical monocytes were defined as CD45+CD11b+CD115+Gr1high cells; Non-classical monocytes were defined as CD45+CD11b+CD115+Gr1Low cells; neutrophils were defined as CD45+CD11b+CD115-Gr1+ cells. Macrophages were defined CD45+CD11b+F4/80+ cells. B, T Lymphocytes were selected as CD45+CD3+ cells. CD4+ T cells were selected as CD45+CD3+CD4+ cells. CD8+ T cells were selected as CD45+CD3+CD8+ cells. B cells were defined as B220+IgM+ cells. C, quantification of spleen leukocyte subsets in the 2 groups (N=10/group). Data are presented as mean values +/- SD. Two-tailed Mann-Whitney test. Source data are provided as a Source Data file.

Supplementary figure 7

Blood

A

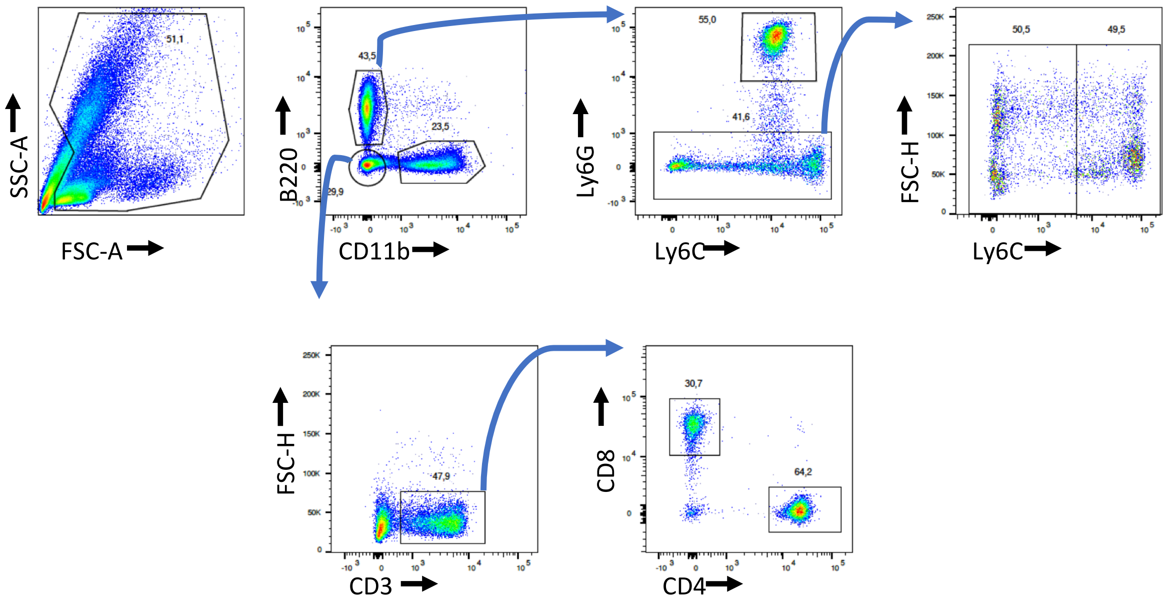

B

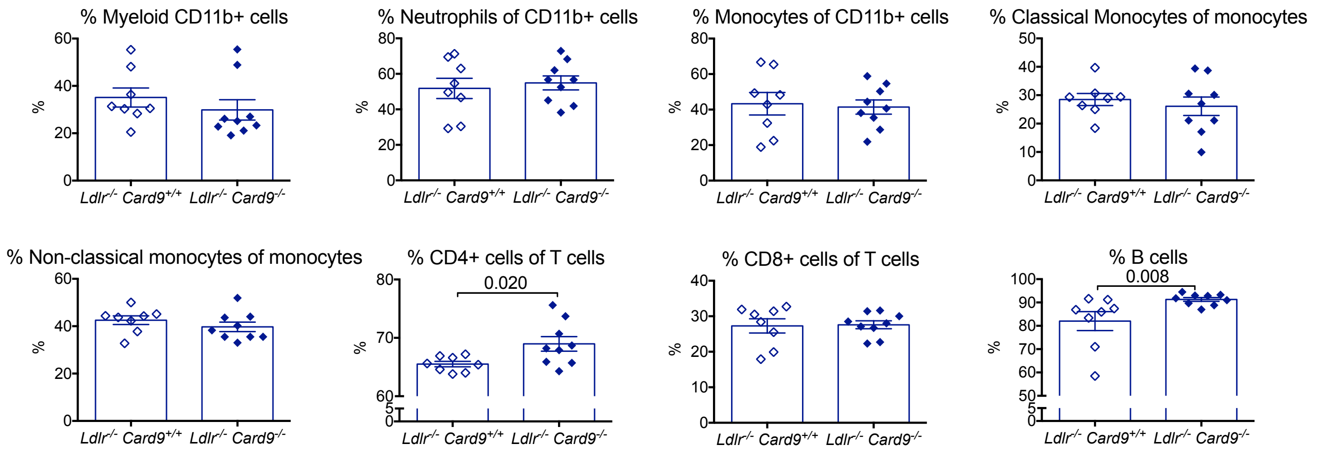

Characterization by flow cytometry of immune cell subsets in the blood of *chimeric $Ldlr^{-/-}Card9^{+/+}$ and $Ldlr^{-/-}Card9^{-/-}$* mice A, classical monocytes were defined as CD11b+Ly6G-Ly6high cells; Non-classical monocytes were defined as CD11b+ Ly6G-Ly6CLow cells; neutrophils were defined as CD11b+ Ly6G+Ly6C- cells. CD4+ T Lymphocytes were selected as B220-CD11b-CD3+CD4+ cells, CD8+ T Lymphocytes were selected as B220-CD11b-CD3+CD8+ cells. B cells were defined as CD11b-B220+ cells. B, quantification of blood leukocyte subsets in the 2 groups (N=8 *$Ldlr^{-/-}Card9^{+/+}$* and N=9 *$Ldlr^{-/-}Card9^{-/-}$*). Data are presented as mean values +/- SD. Two-tailed Mann-Whitney test. Source data are provided as a Source Data file.

Supplementary figure 8

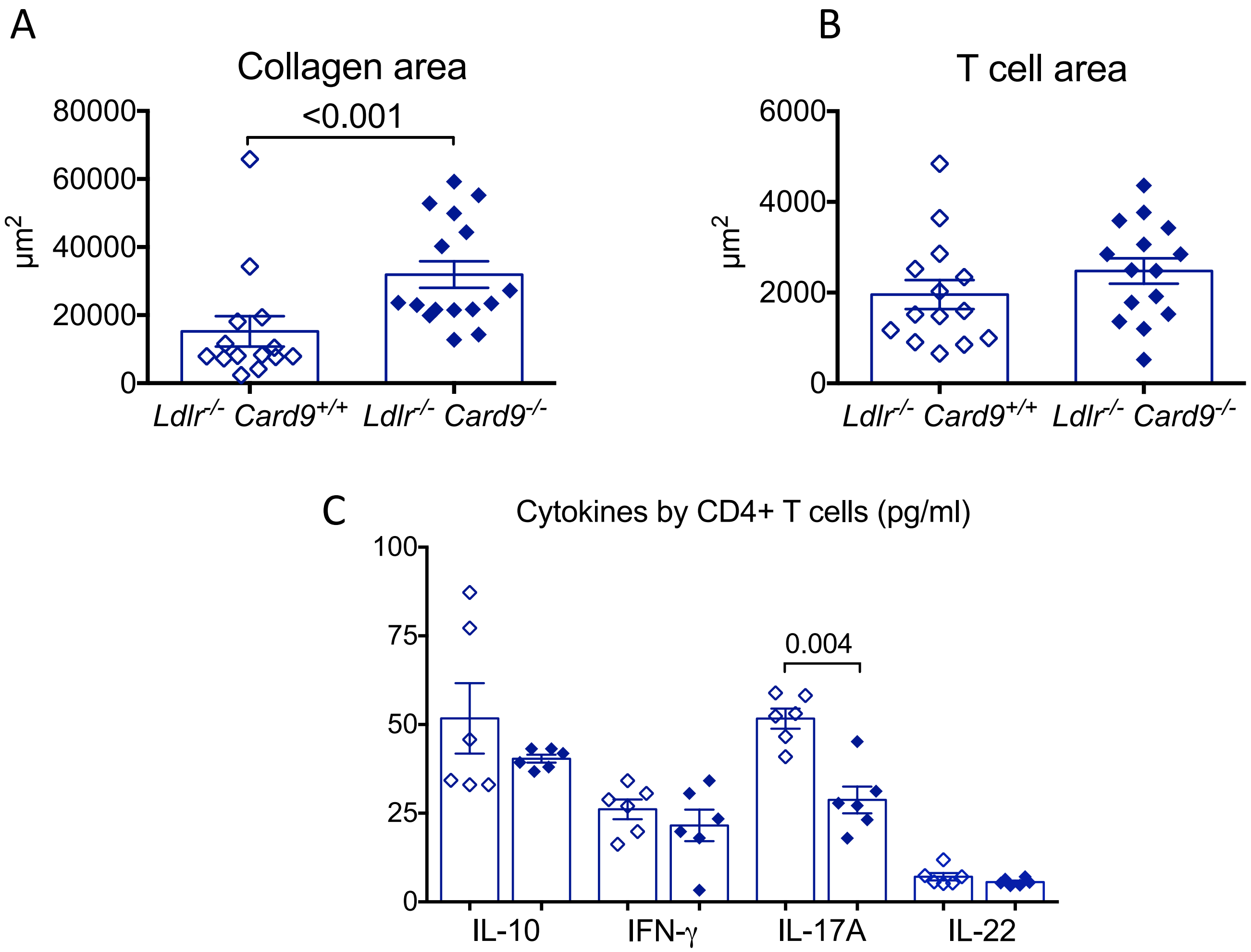

Characterization of chimeric *Ldlr*$^{-/-}$*Card9*$^{+/+}$ and *Ldlr*$^{-/-}$*Card9*$^{-/-}$ mice after 8 weeks of fat diet. A, Quantitative analysis of collagen content (Sirius red) (N=14 *Ldlr*$^{-/-}$*Card9*$^{+/+}$ and N=16 *Ldlr*$^{-/-}$*Card9*$^{-/-}$). B, Quantitative analysis of CD3+ T cell infiltration (Anti-CD3 immunostaining) (N=14 *Ldlr*$^{-/-}$*Card9*$^{+/+}$ and N=16 *Ldlr*$^{-/-}$*Card9*$^{-/-}$). C, cytokine production (ELISA) of purified splenic CD4+ T cells from chimeric Ldlr-/-Card9+/+ and Ldlr-/-Card9-/- animals after 48 hours of coated anti-CD3 stimulation (n=6/group). Data are presented as mean values +/- SD. Two-tailed Mann-Whitney test. Source data are provided as a Source Data file.

Supplementary figure 9

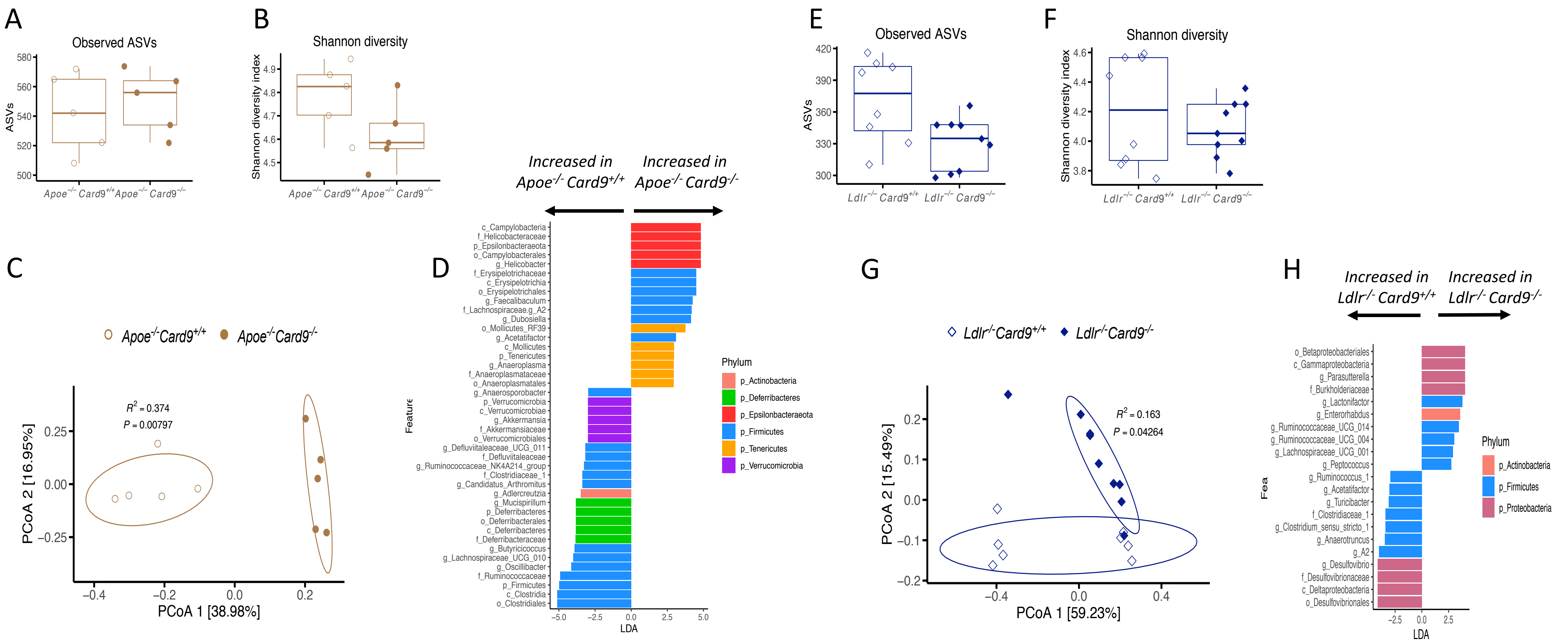

Effects of *Card9* deficiency on microbiota composition was not consistent between different models of atherosclerosis. A, Amplicon Sequence Variants (ASV) and (B) Shannon index evaluate alpha diversity (N=5/group). C, principal component analysis (PCoA) based on bacterial 16S ribosomal DNA gene sequence abundance in fecal content of *Apoe*$^{-/-}$*Card9*$^{+/+}$and *Apoe*$^{-/-}$*Card9*$^{-/-}$ mice fed with high fat diet. D, Bacterial-taxon-based analysis in the fecal microbiota. E, Amplicon Sequence Variants (ASV) and (F) Shannon index evaluate alpha diversity (N=8 *Ldlr*$^{-/-}$*Card9*$^{+/+}$ and N=9 *Ldlr*$^{-/-}$*Card9*$^{-/-}$). G, principal component analysis (PCoA) based on bacterial 16S ribosomal DNA gene sequence abundance in fecal content of chimeric *Ldlr*$^{-/-}$*Card9*$^{+/+}$ and *Ldlr*$^{-/-}$*Card9*$^{-/-}$ mice fed with high fat diet. H, Bacterial-taxon-based analysis in the fecal microbiota. A-B, E-F, Box showed median value (1st IQR-3rd IQR) and whiskers (5-95 percentiles). C, G, PERMANOVA non parametric multivariate test.

Supplementary figure 10

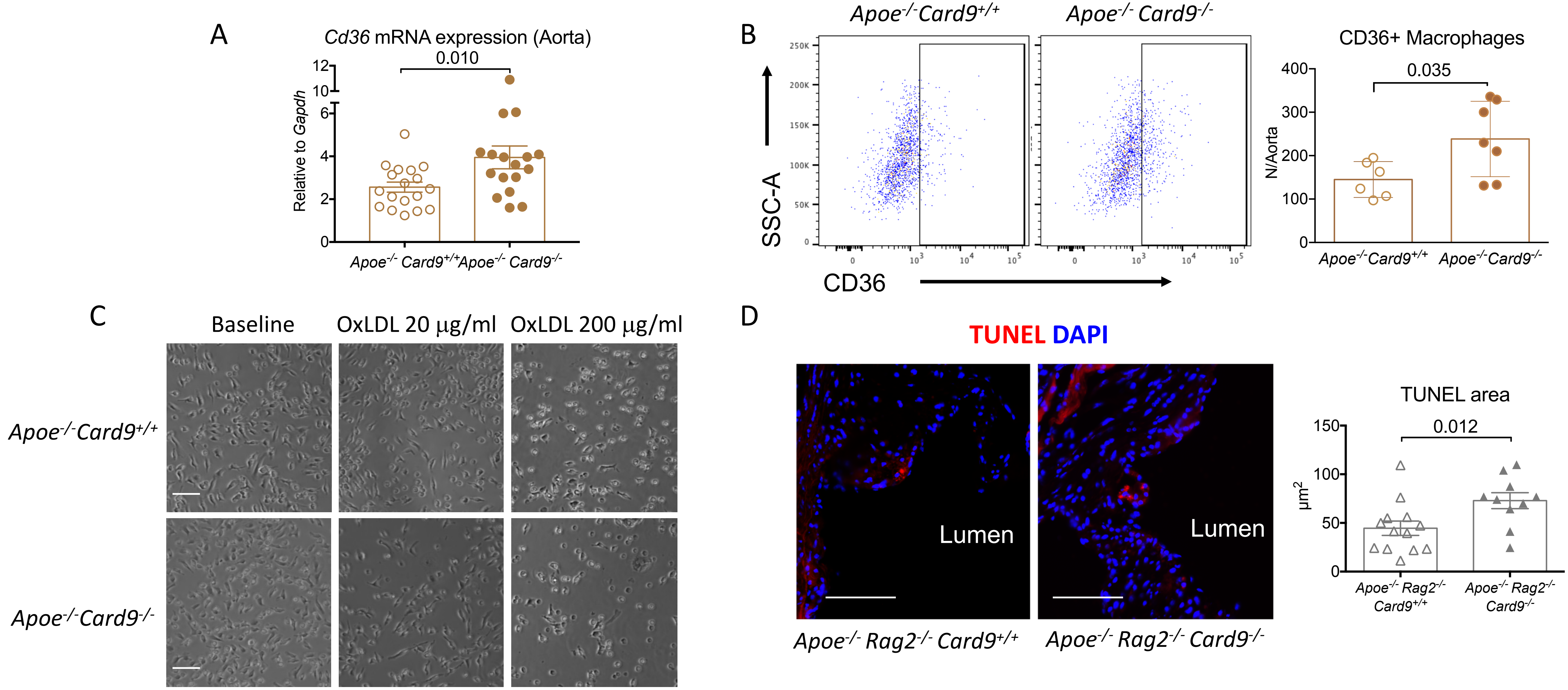

***Card9* deficiency increased cell death susceptibility.** A, *Cd36* mRNA quantification by qPCR in the thoraco-abdominal aorta of *Apoe*$^{-/-}$*Card9*$^{+/+}$ and *Apoe*$^{-/-}$*Card9*$^{-/-}$mice after 6 weeks of fat diet (N=18 *Apoe*$^{-/-}$*Card9*$^{+/+}$ and N=17 *Apoe*$^{-/-}$*Card9*$^{-/-}$). B, CD36 expression evaluated by flow cytometry on CD45+CD11B+Ly6G-CD64+ macrophages within the aorta of *Apoe*$^{-/-}$*Card9*$^{+/+}$ and *Apoe*$^{-/-}$*Card9*$^{-/-}$mice after 6 weeks of fat diet (Representative flow pictures of N=6 *Apoe*$^{-/-}$*Card9*$^{+/+}$ and N=7 *Apoe*$^{-/-}$*Card9*$^{-/-}$ *group*). C, BM-derived macrophages from *Apoe*$^{-/-}$*Card9*$^{+/+}$ and *Apoe*$^{-/-}$*Card9*$^{-/-}$mice were stimulated in vitro by oxLDL during 12 hours and density was evaluated (3 pooled experiments, n=9/condition), Scale bar 20 μm. D, representative photomicrographs and quantitative analysis of TUNEL staining in atherosclerotic lesions of *Apoe*$^{-/-}$ *Rag2*$^{-/-}$*Card9*$^{+/+}$ (N=13) *and Apoe*$^{-/-}$ *Rag2*$^{-/-}$ *Card9*$^{-/-}$ mice (N=10). Scale bar 20 μm. Data are presented as mean values +/- SD. Two-tailed Mann-Whitney test. Source data are provided as a Source Data file.

Supplementary figure 11

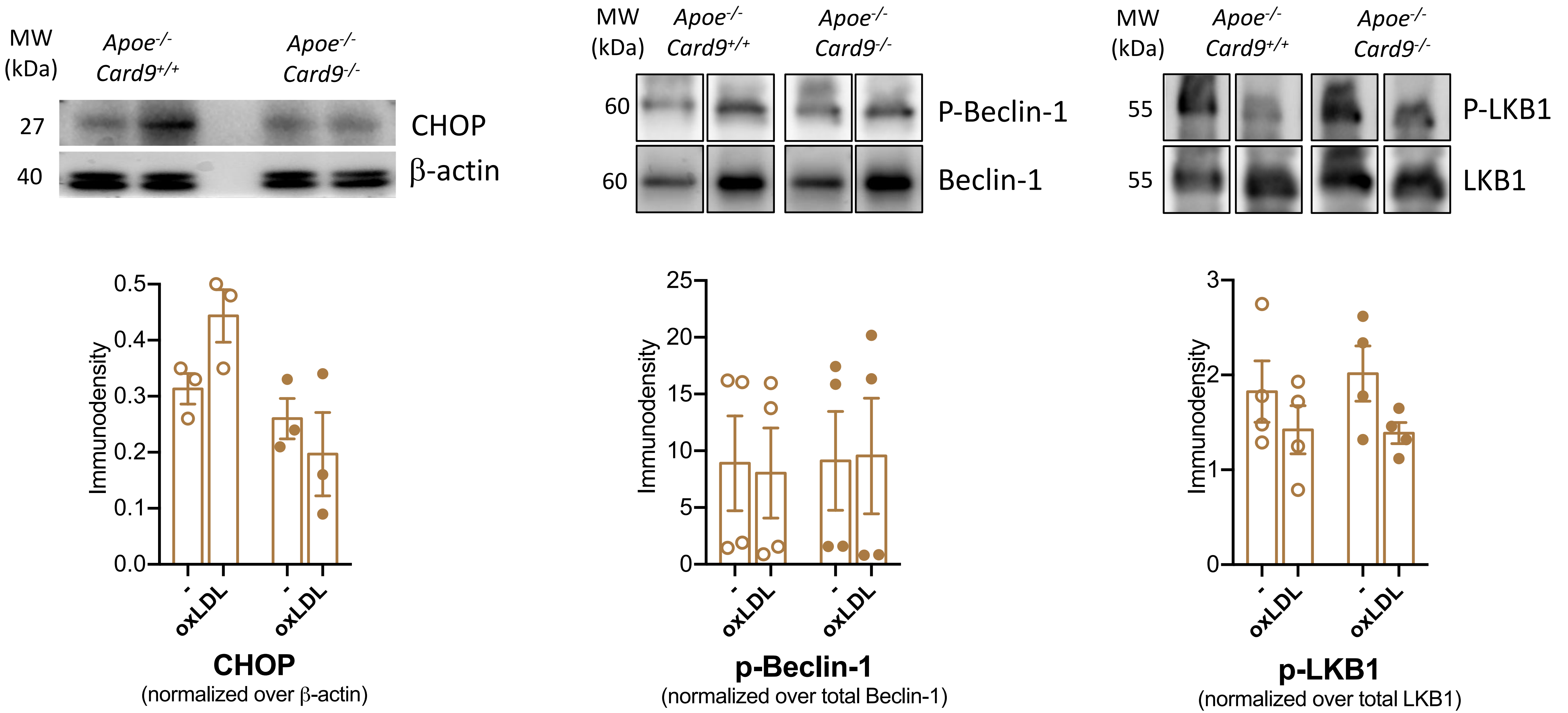

Dowstream autophagy pathways in macrophages. Bone marrow-derived macrophages isolated from *Apoe*$^{-/-}$*Card9* $^{+/+}$ or *Apoe*$^{-/-}$*Card9* $^{+/+}$ mice were challenged *in vitro* during 8 hours with Ox-LDL (50 µg/mL). CHOP as well as phosphorylation of Beclin and LKB1 were quantified by Western Blot (N=4/group/condition). Data are presented as mean values +/- SD.

Supplementary figure 12

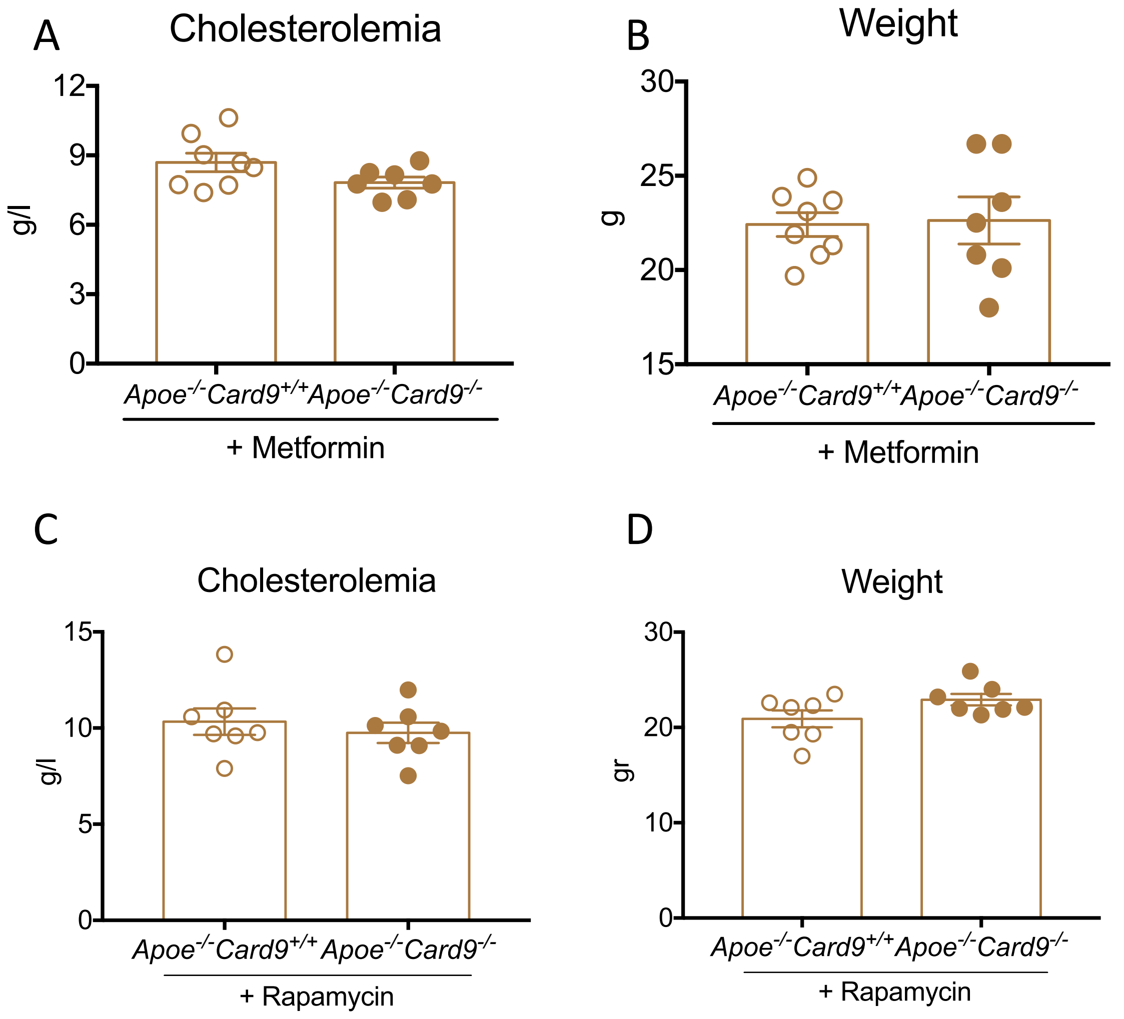

Pharmacological modulation of autophagy in vivo. 8-week old *Apoe$^{-/-}$Card9$^{+/+}$ and Apoe$^{-/-}$Card9$^{-/-}$* mice were treated with metformin (300mg/kg/day, drinking water) and put under a high fat diet during 6 weeks (n=8 *Apoe$^{-/-}$Card9$^{+/+}$* and N=7 *Apoe$^{-/-}$Card9$^{-/-}$*). Plasma cholesterol level (A) and body weight (B) at sacrifice. 8-week old *Apoe$^{-/-}$Card9$^{+/+}$ and Apoe$^{-/-}$Card9$^{-/-}$* mice were treated IP with rapamycin (4 mg/kg/day) and were put under a high fat diet during 6 weeks. C, plasma cholesterol level (n=7/group) and (D) body weight at sacrifice (n=7/group). Data are presented as mean values +/- SD.

Supplementary figure 13

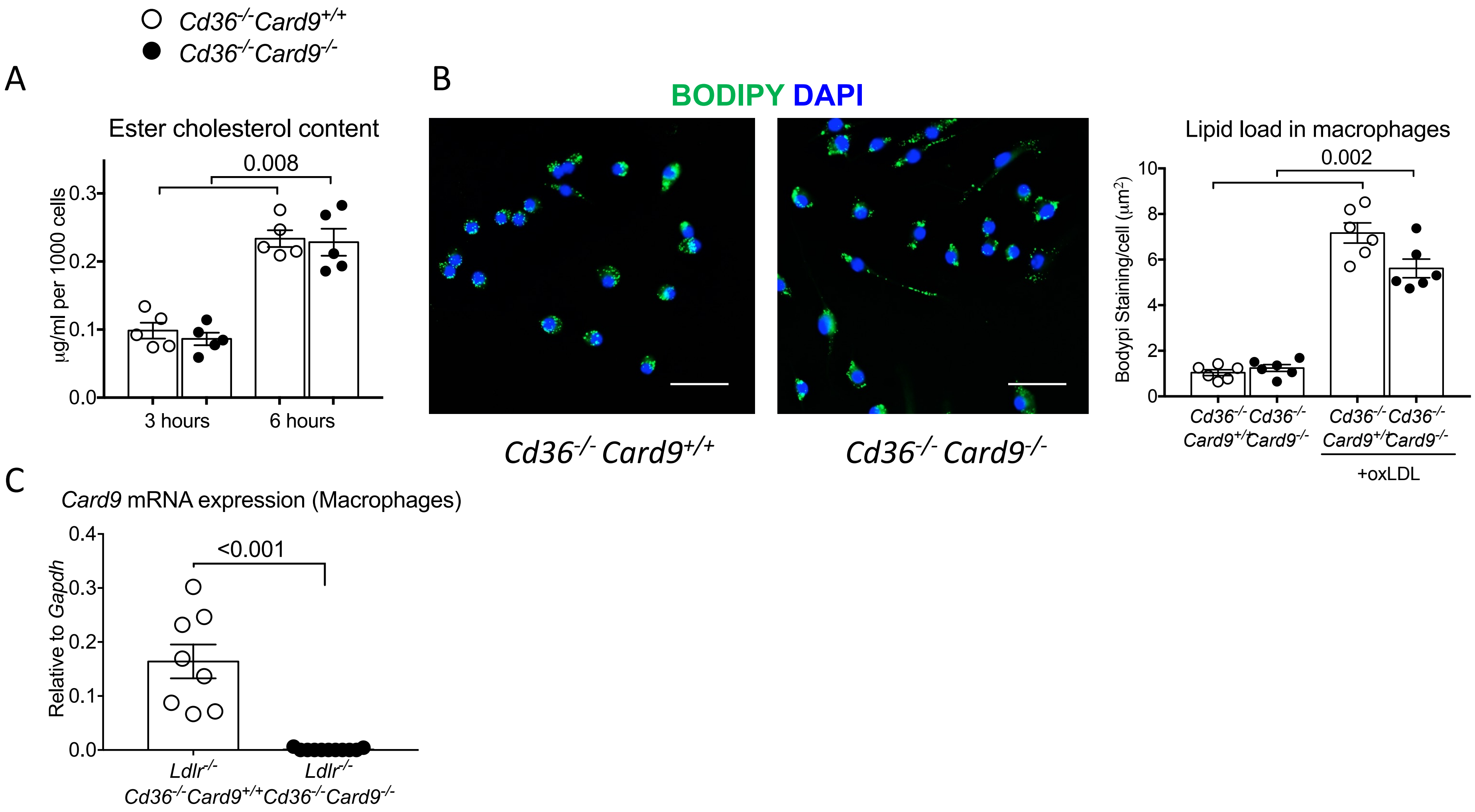

Lipid uptake in the absence of CD36. A, quantification of intracellular ester cholesterol on BM-derived macrophages from *cd36*$^{-/-}$*Card9*$^{+/+}$ and *Cd36*$^{-/-}$*Card9*$^{-/-}$ mice after exposure to ox-LDL (n=5/group/timepoints). B, Representative photomicrographs and quantitative analysis of Bodipy+ foam cells (green) after incubation of BM-derived macrophages from *CD36*$^{-/-}$*Card9*$^{+/+}$ and *Cd36*$^{-/-}$*Card9*$^{-/-}$ mice with oxLDL during 24 hours (n=6/group/timepoints), Scale bar 10 μm. C, *Card9* mRNA expression in peritoneal macrophages of chimeric *Ldlr*$^{-/-}$*Cd36*$^{-/-}$*Card9*$^{+/+}$ and *Ldlr*$^{-/-}$*Cd36*$^{-/-}$*Card9*$^{-/-}$ mice (n=8/group). Data are presented as mean values +/- SD. Two-tailed Mann-Whitney test. Source data are provided as a Source Data file.

Supplementary figure 14

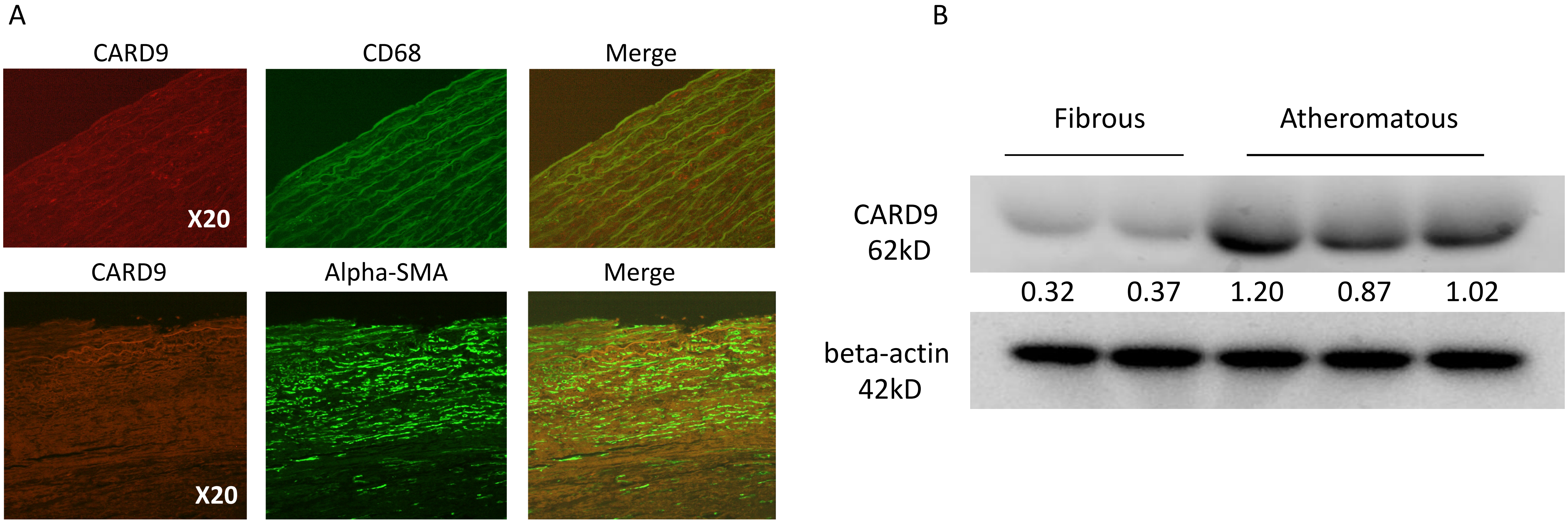

CARD9 in human tissue . A, CARD9 protein expression in human normal aorta using immunofluorescent staining**.** CARD9 (Red) is not expressed in normal aorta. CD68 (Green) is a marker of macrophages and Alpha-SMA is a marker of smooth muscle cells. Magnitude X20 (2 pooled experiments, n=6/staining). B, CARD9 quantification in fibrous and atheromatous human plaques by Western Blot. CARD9 expression relative to beta-actin (1 experiment, n=2 fibrous and n=3 atheromatous).

Supplementary figure 15

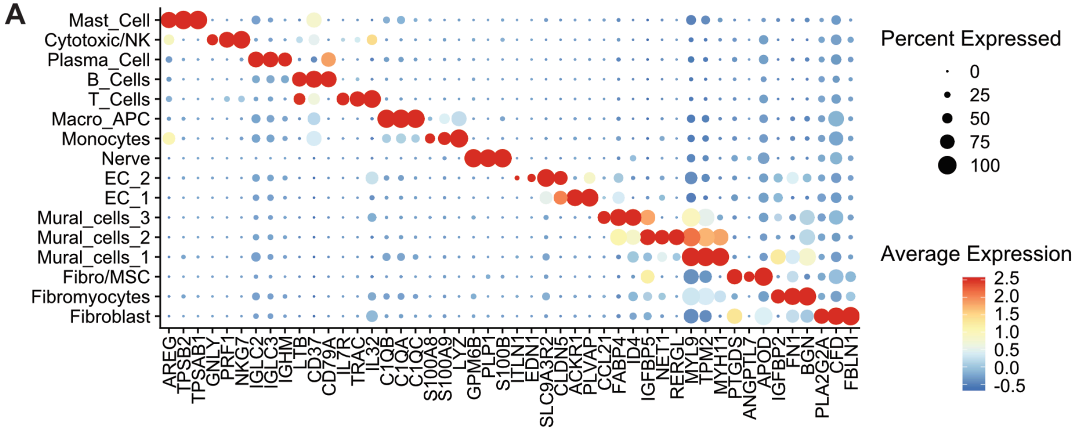

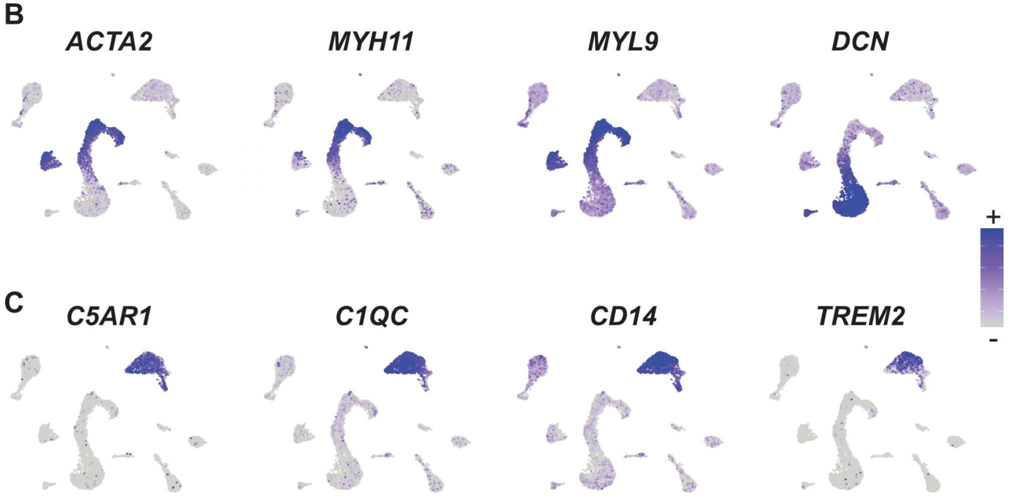

D

	% CARD9+
Fibroblasts	0.2
Fibromyocytes	0.3
Fibro/MSC	0.0
Mural_cells_1	0.0
Mural_cells_2	0.0
Mural_cells_3	0.2
EC_1	0.2
EC_2	0.0
Nerve	0.0
Monocytes	19.3
Macro_APC	13.6
T_Cells	0.2
B_Cells	0.2
Plasma_Cell	0.5
Cytotoxic/NK	0.0
Mast_Cell	2.3

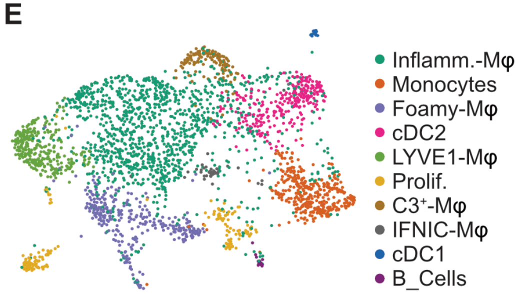

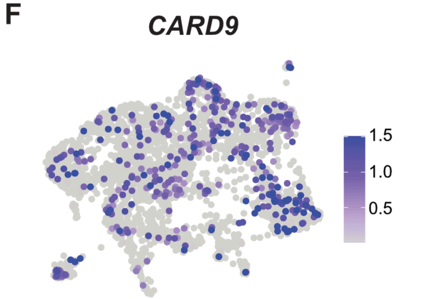

G

	% CARD9+
LYVE1_Mac	17.8
Inflamm_Mac	17.0
Foamy_Mac	15.3
IFNIC_Mac	21.4
C3_Mac	25.0
Monocytes	19.6
Prolif	29.6
cDC1	17.4
cDC2	26.2

Single-cell RNA-seq analysis of *CARD9* expression in human atherosclerosis. A, dot plot showing average expression of the top3 marker transcripts (ranked by fold change) in each cluster in the analysis from **Figure 8F**; B, expression of mural cell (*ACTA2, MYH11, MYL9*) and fibroblast (*DCN*) markers projected onto the UMAP plot; C, expression of macrophage markers (*C5AR1* encoding *CD88, C1QC, CD14, TREM2*) projected onto the UMAP plot. In B and C, expression cutoffs have been applied. D, proportion of cells with detectable *CARD9* transcripts across clusters. E, UMAP representation and clustering analysis of integrated scRNA-seq gene expression data in 2,890 human mononuclear phagocytes from atherosclerotic lesions, panel is reproduced from Zernecke et al. *Cardiovascular Research* 2022; F, expression of *CARD9* projected onto the UMAP plot, minimum and maximum gene expression cutoffs were applied, and cells with detectable transcripts were brought to the front of plots using the “order=TRUE” argument within the Seurat FeaturePlot function.; G, proportion of cells with detectable CARD9 transcripts across mononuclear phagocyte clusters.

Supplementary figure 16

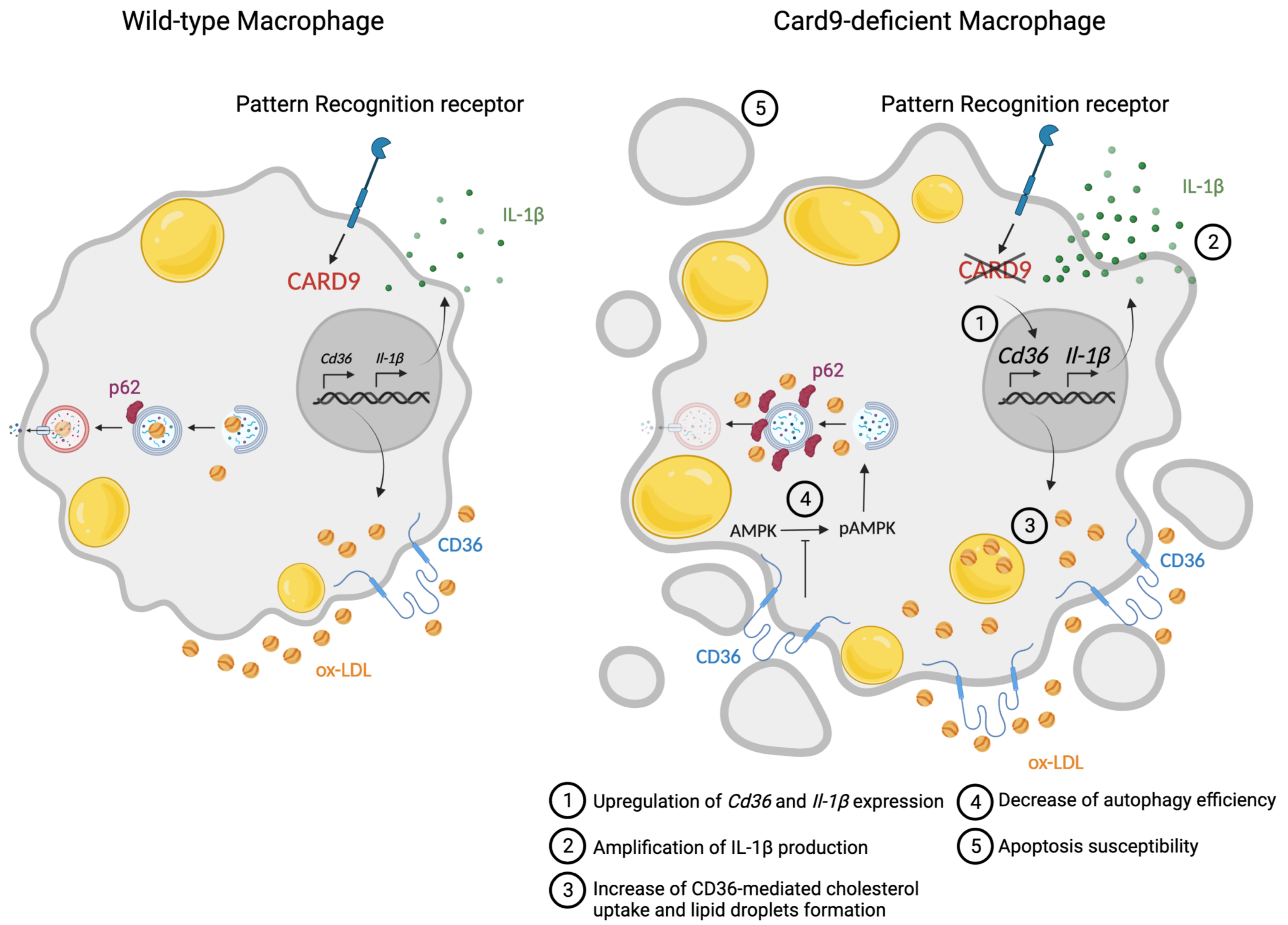

Summary cartoon describing the pro-atherogenic mechanisms of Card9 deficiency

Uncropped Western blot

Figure 5A

P-AMPK

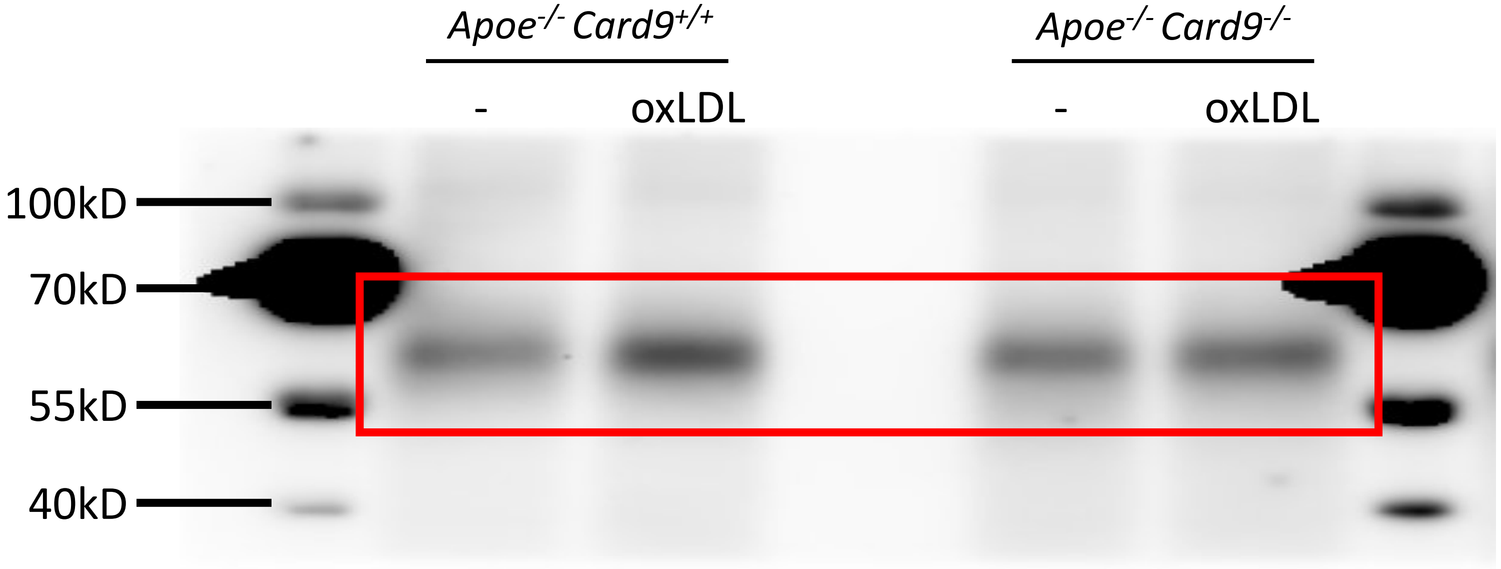

Figure 5A

AMPK

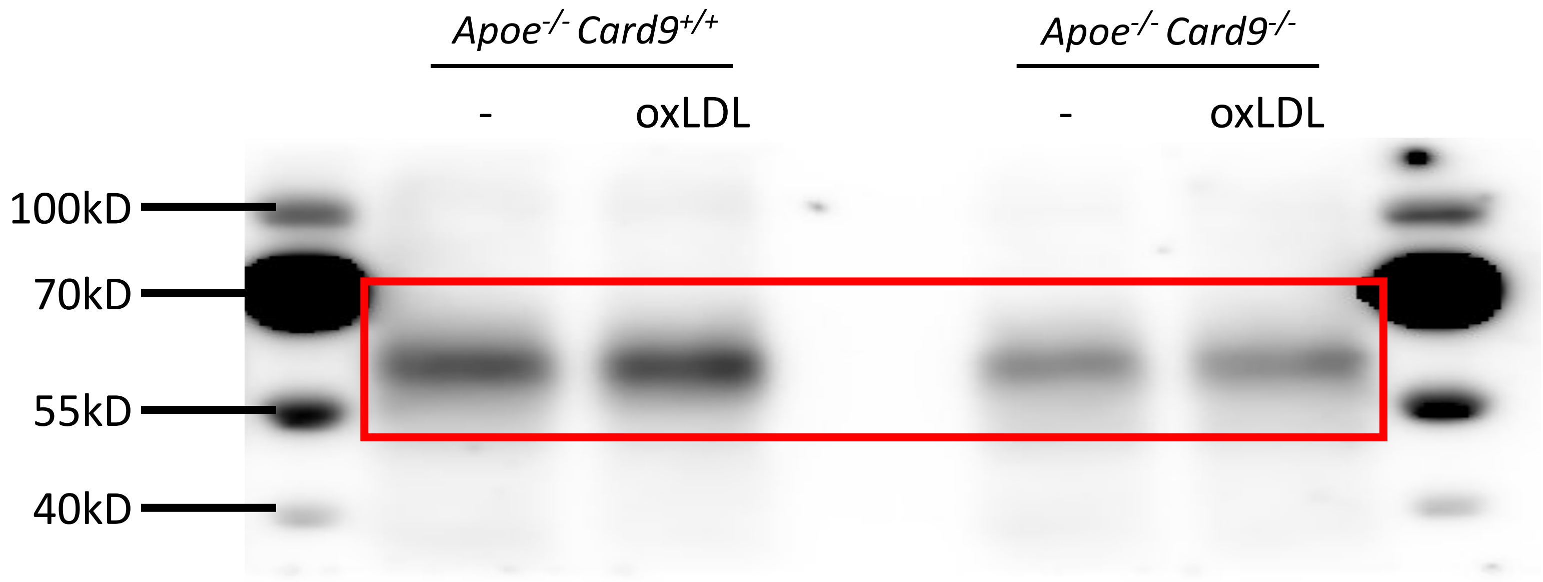

Figure 5A

P62

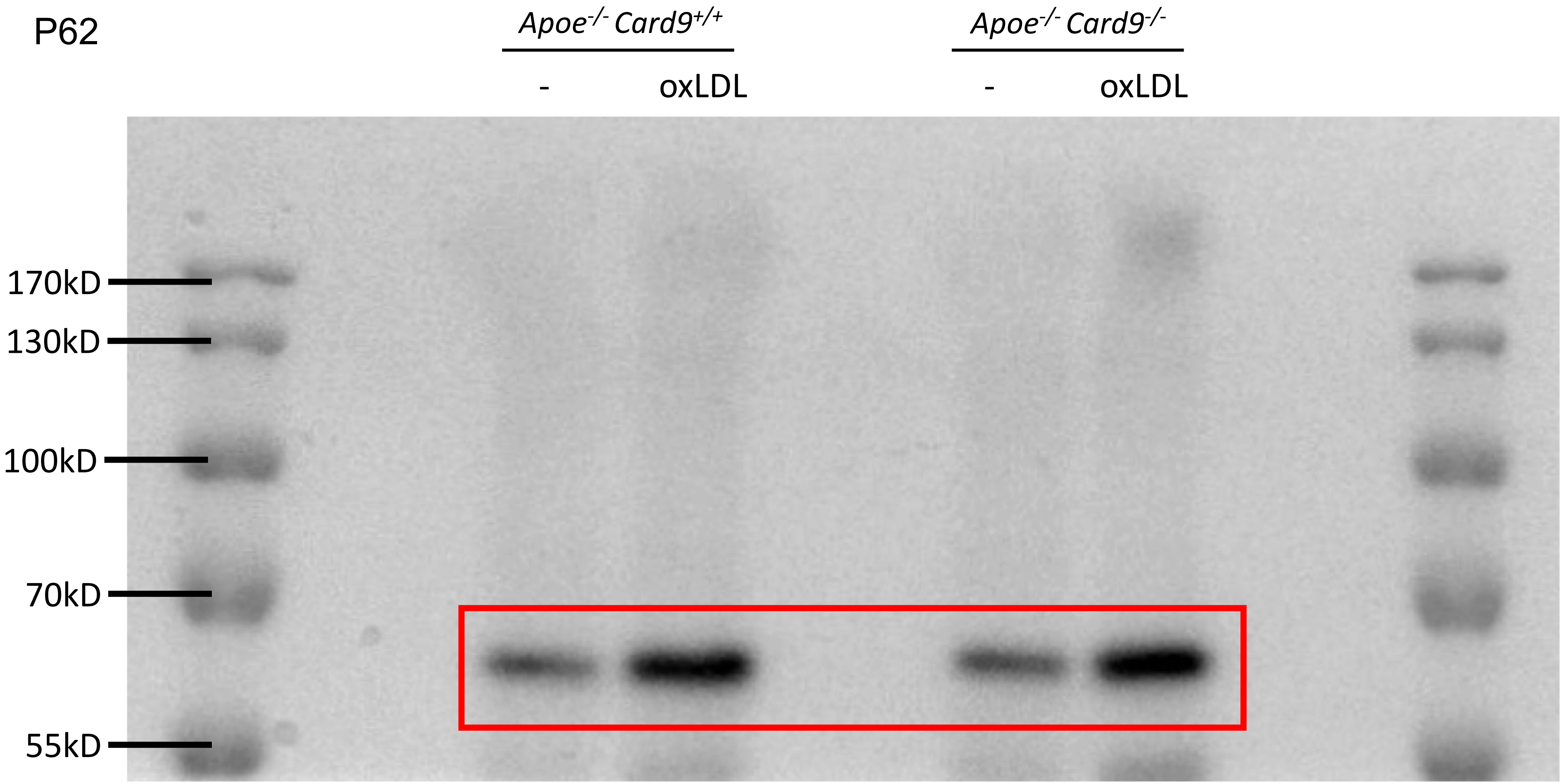

Figure 5A

b-actin

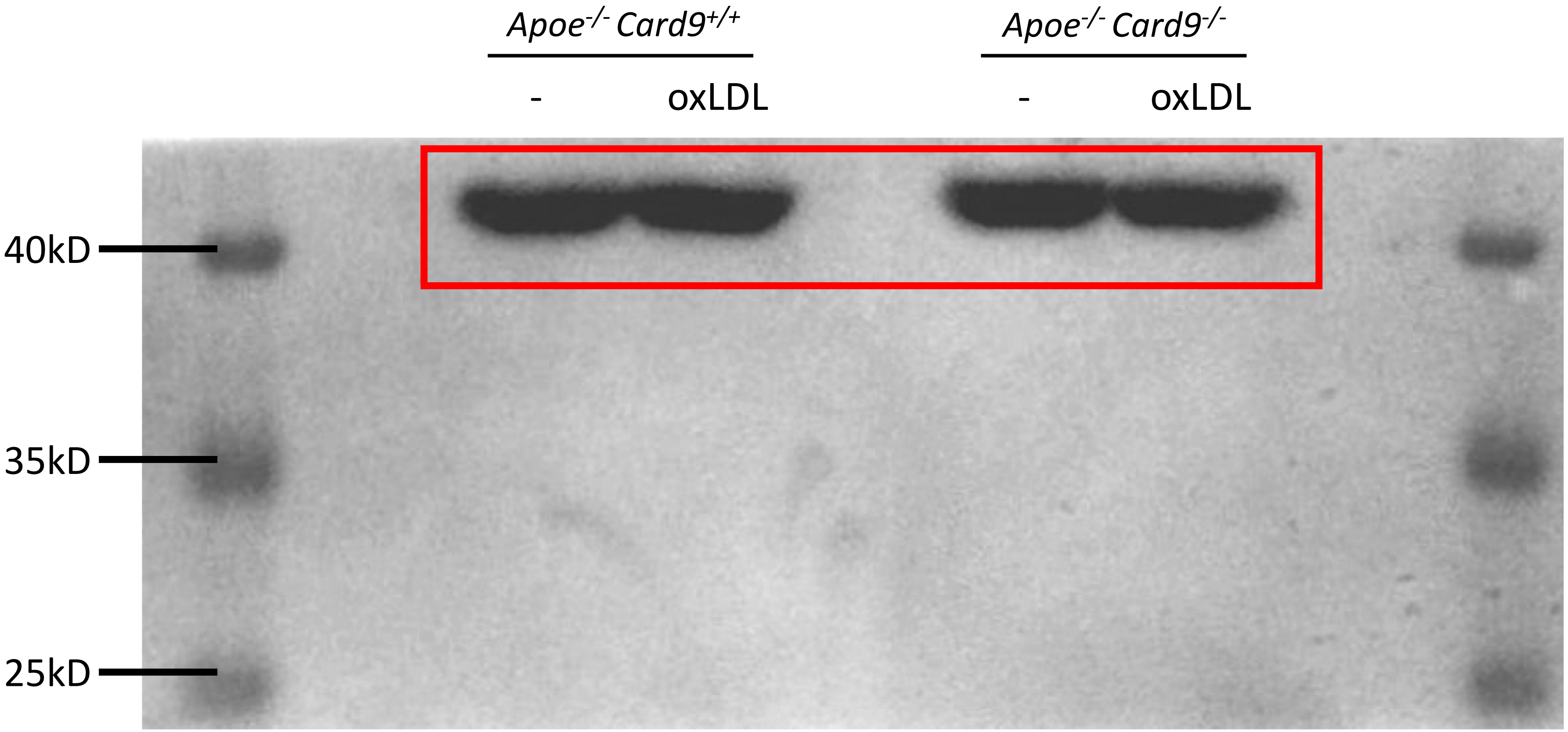

Figure 7A

p-AMPK

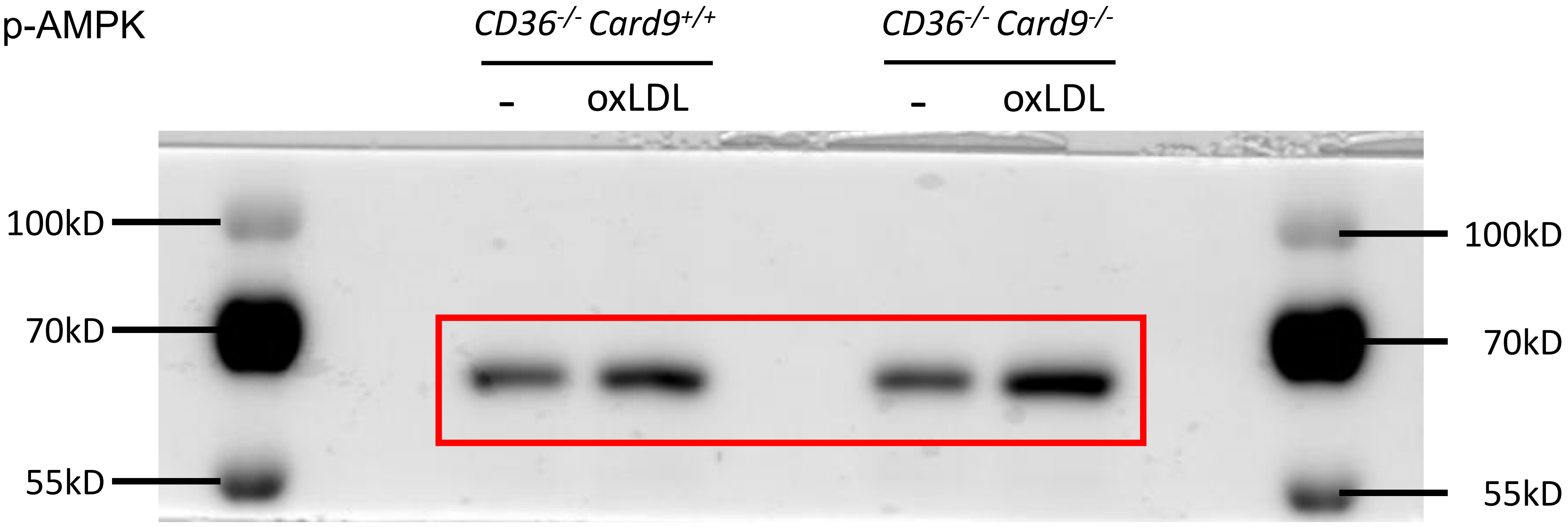

Figure 7A

AMPK

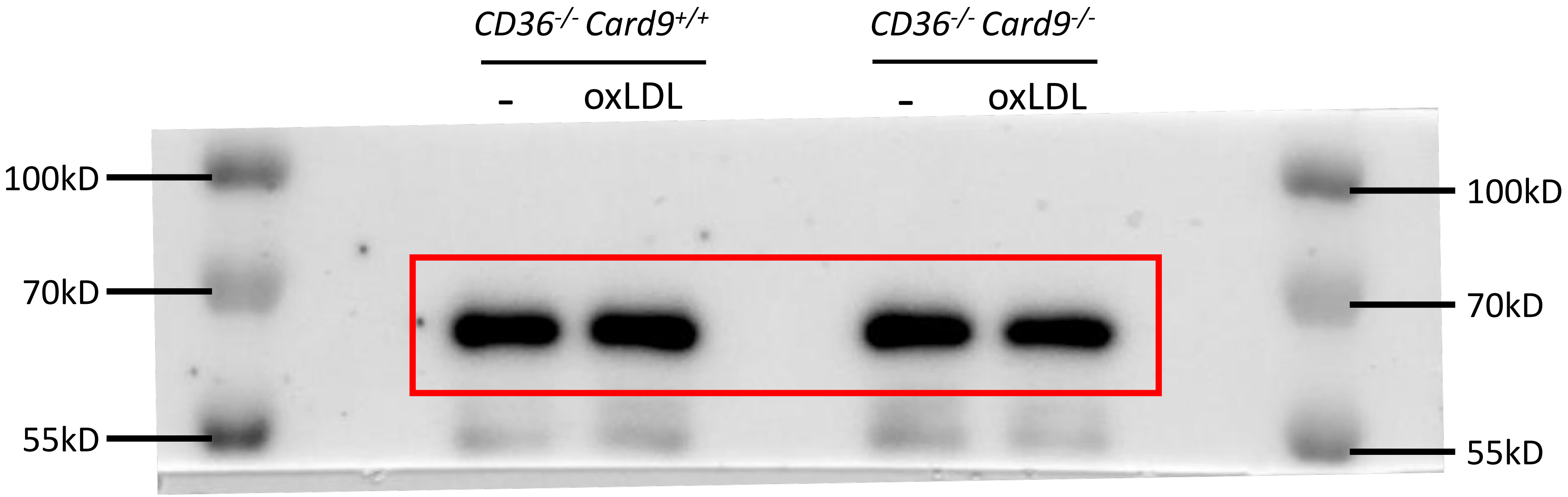

Supplementary figure 11

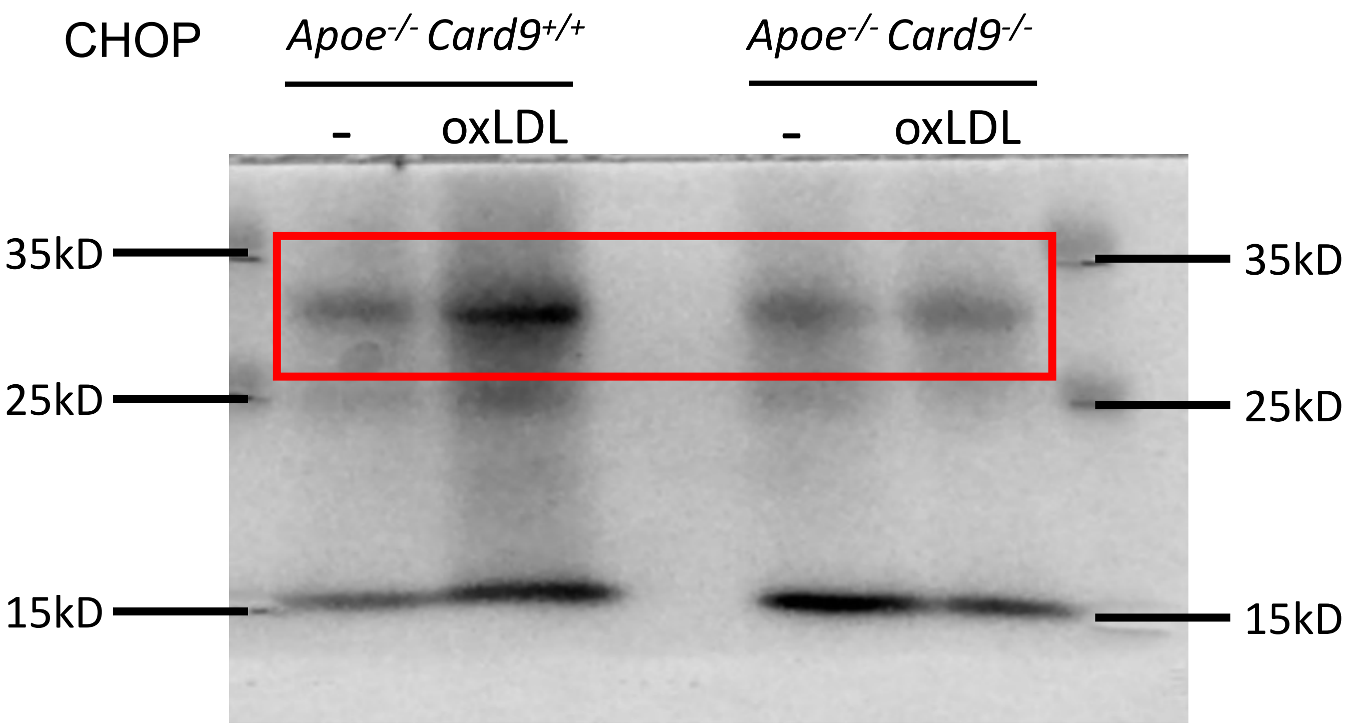

Supplementary figure 11

b-actin

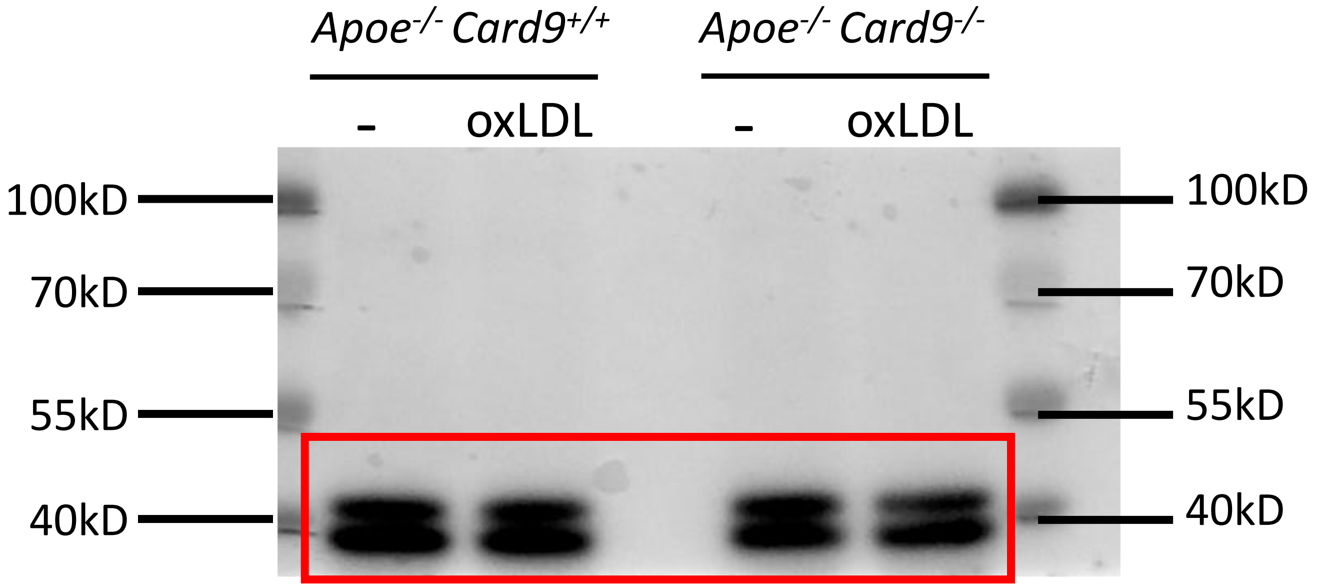

Supplementary figure 11

P-Beclin-1

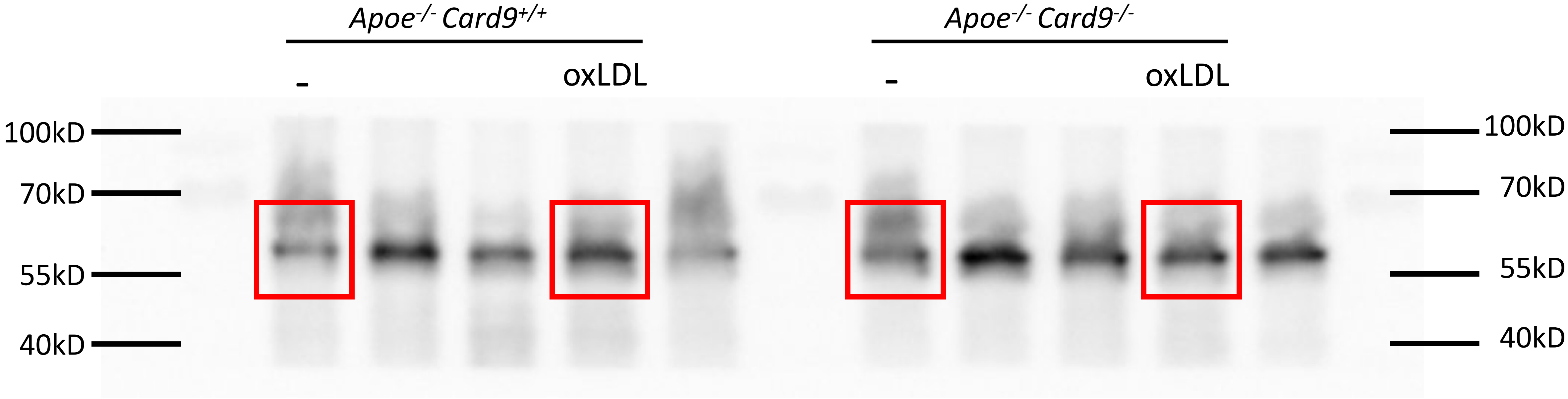

Supplementary figure 11

Beclin-1

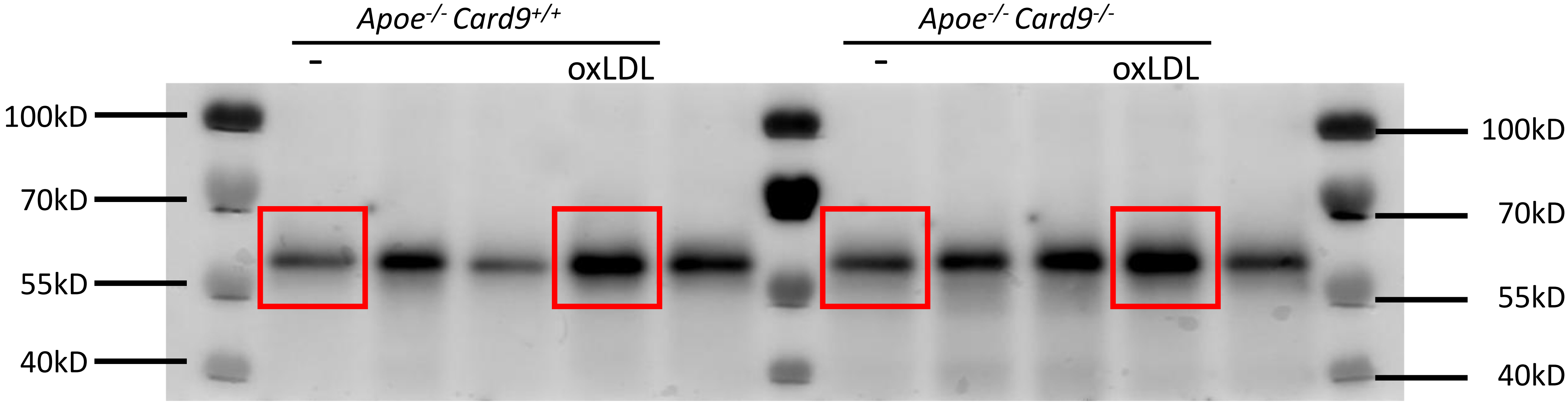

Supplementary figure 11

P-LKB1

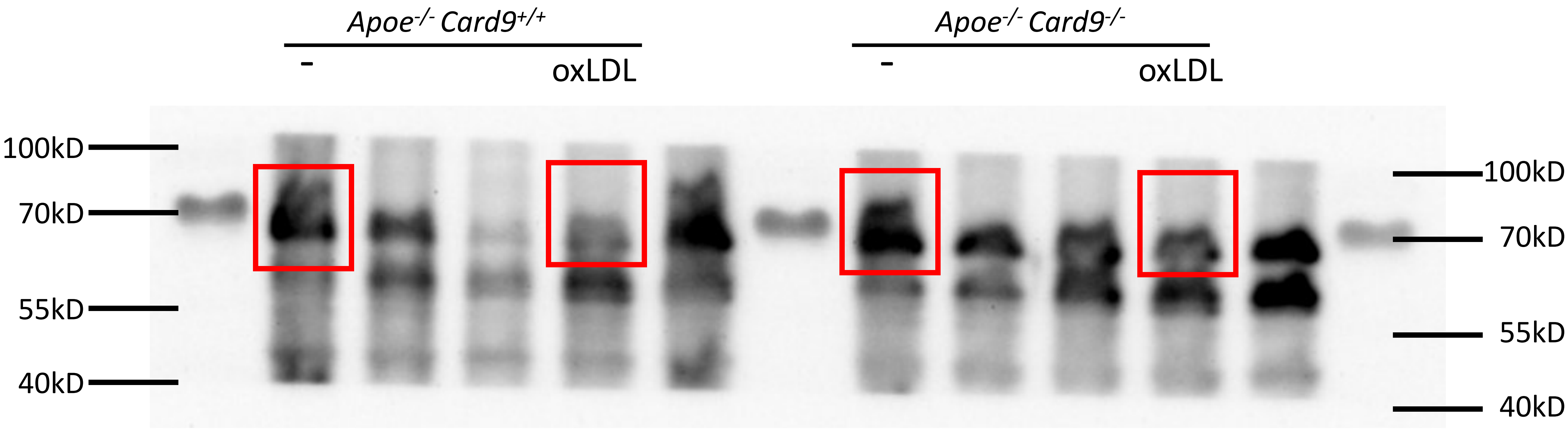

Supplementary figure 11

LKB1

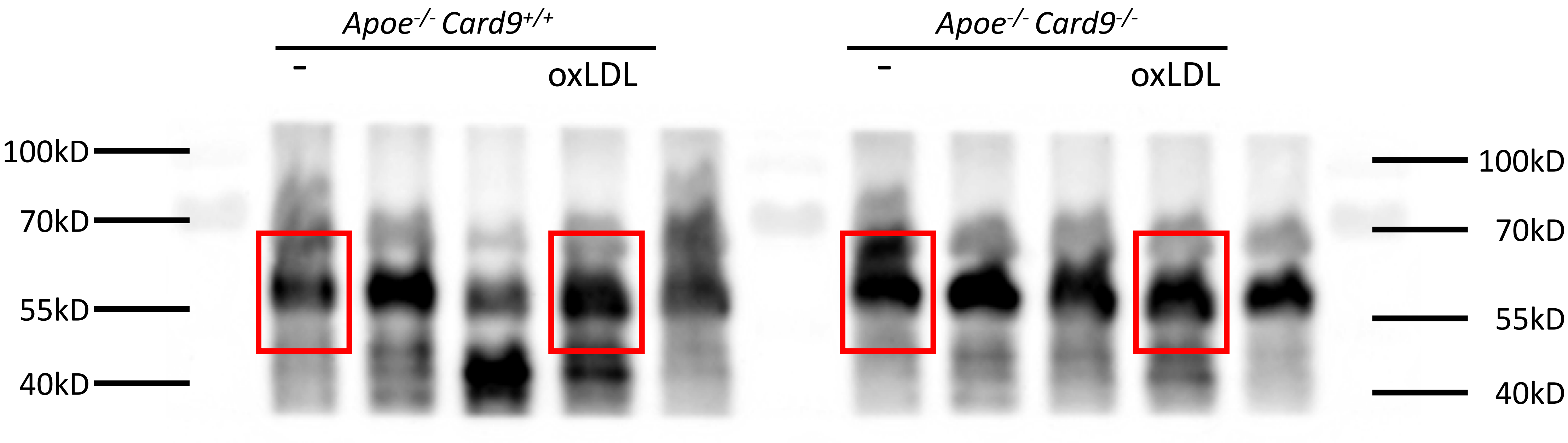

Supplementary figure 14B

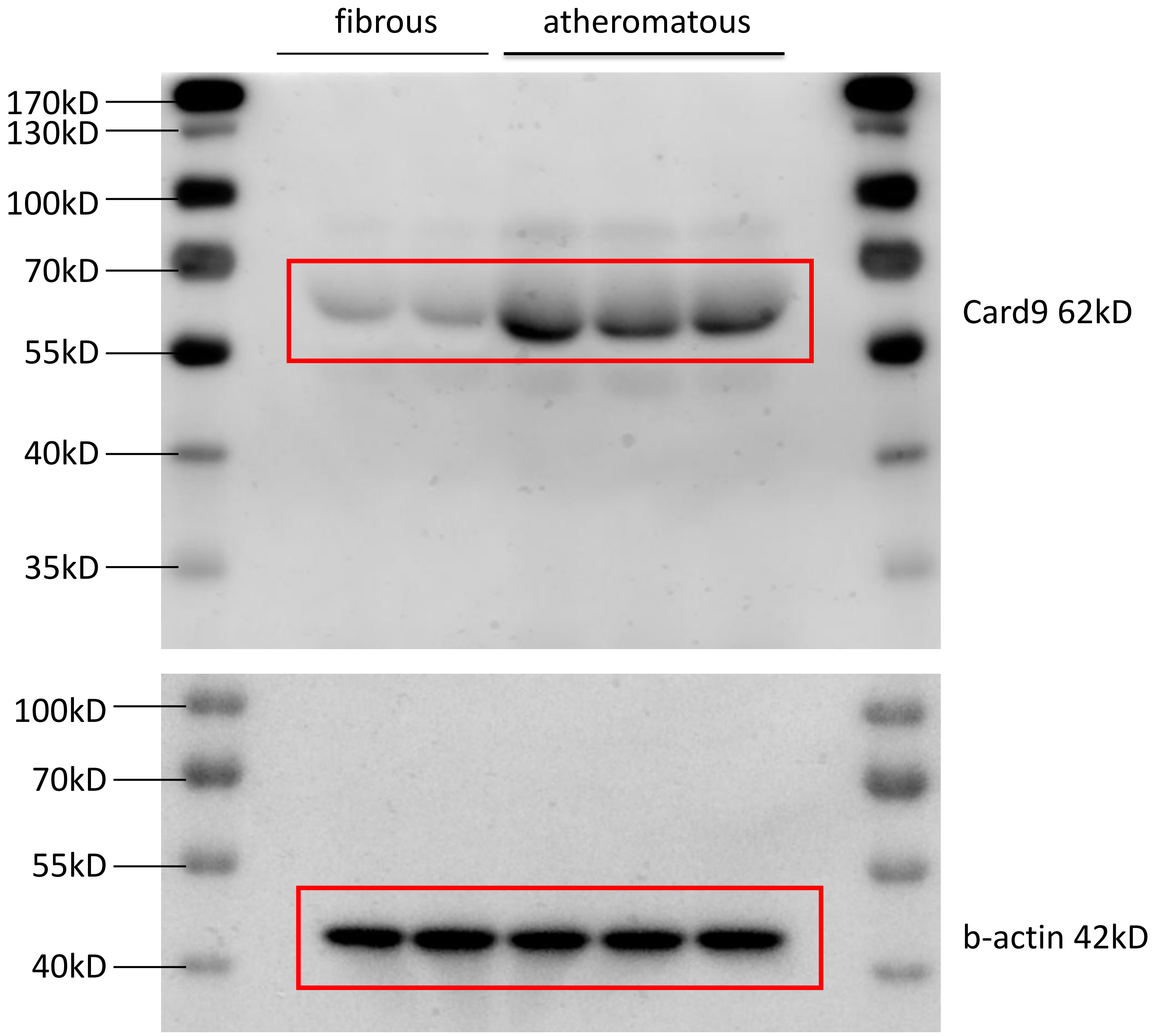

Supplement: Supplementary file 1 — Supplementary Information [file 41467_2023_40216_MOESM1_ESM.pdf]
