# Supplementary material for: Genetic inhibition of CARD9 accelerates the development of atherosclerosis in mice through CD36 dependent-defective autophagy
Source: Nat Commun. 2023 Aug 1;14:4622. doi: 10.1038/s41467-023-40216-x (PMC10394049; doi:10.1038/s41467-023-40216-x)
Supplement: Supplementary file 3 — Description of Additional Supplementary Files [file 41467_2023_40216_MOESM3_ESM.pdf]

<u>Description of Additional Supplementary Files</u>

Supplementary data 1a : RLE Row and normalized counts

Supplementary data 1b : Row and normalized counts

Supplementary data 2: selected pathways and core enrichment genes; Statistics computed by GSEA

Supplementary data 3: List of antibodies used in the study
