# Supplementary material for: Genetic inhibition of CARD9 accelerates the development of atherosclerosis in mice through CD36 dependent-defective autophagy
Source: Nat Commun. 2023 Aug 1;14:4622. doi: 10.1038/s41467-023-40216-x (PMC10394049; doi:10.1038/s41467-023-40216-x)
Supplement: Supplementary file 5 — Reporting Summary [file 41467_2023_40216_MOESM5_ESM.pdf]

nature portfolio

Corresponding author(s): Ait-Oufella

Last updated by author(s): Jul 12, 2023

Reporting Summary

Nature Portfolio wishes to improve the reproducibility of the work that we publish. This form provides structure for consistency and transparency in reporting. For further information on Nature Portfolio policies, see our Editorial Policies and the Editorial Policy Checklist.

Statistics

For all statistical analyses, confirm that the following items are present in the figure legend, table legend, main text, or Methods section.

n/a	Confirmed	
☐	☒	The exact sample size (*n*) for each experimental group/condition, given as a discrete number and unit of measurement
☐	☒	A statement on whether measurements were taken from distinct samples or whether the same sample was measured repeatedly
☐	☒	The statistical test(s) used AND whether they are one- or two-sided<br>*Only common tests should be described solely by name; describe more complex techniques in the Methods section.*
☐	☒	A description of all covariates tested
☐	☒	A description of any assumptions or corrections, such as tests of normality and adjustment for multiple comparisons
☐	☒	A full description of the statistical parameters including central tendency (e.g. means) or other basic estimates (e.g. regression coefficient) AND variation (e.g. standard deviation) or associated estimates of uncertainty (e.g. confidence intervals)
☐	☒	For null hypothesis testing, the test statistic (e.g. *F*, *t*, *r*) with confidence intervals, effect sizes, degrees of freedom and *P* value noted<br>*Give P values as exact values whenever suitable.*
☒	☐	For Bayesian analysis, information on the choice of priors and Markov chain Monte Carlo settings
☒	☐	For hierarchical and complex designs, identification of the appropriate level for tests and full reporting of outcomes
☒	☐	Estimates of effect sizes (e.g. Cohen's *d*, Pearson's *r*), indicating how they were calculated

Our web collection on statistics for biologists contains articles on many of the points above.

Software and code

Policy information about availability of computer code

Data collection	RNA-sequencing data of blood monocytes generated for this report has been deposited in Gene Expression Omnibus (https://www.ncbi.nlm.nih.gov/geo/query/acc.cgi?acc= GSE221782). Mouse scRNA-seq data were downloaded from Gene Expression Omnibus GSE155513 (ref 18), GSM4705592 (ref 18), GSM4705593 (ref 18), GSM4705594 (ref18), GSM4705595 (ref 18), GSM4705596 (ref 18), GSM4705597 (ref 18), GSM4705598 (ref 18), GSM4705599 (ref 18), GSE131780 (ref 33). Microbiota 16s RNA data are accessible with the following link https://www.ncbi.nlm.nih.gov/sra/PRJNA986053. Human carotid atherosclerosis scRNA-seq data were downloaded from https://figshare.com/s/c00d88b1b25ef0c5c788 (ref 71). Human coronary atherosclerosis scRNA-seq data were downloaded from Gene Expression Omnibus GSE131776 (ref 33) & GSE131778 (ref 33).
Data analysis	For transcriptomic analysis on human monocytes, We performed the gene set enrichment analysis using clusterProfiler v4.0.5 54 with selected pathways from Gene Ontology (GO) and Kyoto Encyclopedia of Genes and Genomes (KEGG) databases, and Benjamini-Hochberg correction was applied. Used keywords were: “apopto*”, “atheroscleros*”, “NF-kappa B”, “TNF”. For single-cell analysis of Card9/CARD) expression patterns, cells containing >200 detected genes, and genes detected in at least 3 cells were included in the analysis using the ‘CreateSeuratObject’ function with ‘min.features = 200’ and ‘min.cells=3’. Quality control filtering was further performed to remove dead/damaged cells with a high proportion of mitochondrial transcripts, and outlier cells with high UMI numbers (probable doublets/mutliplets). For mitochondrial transcripts, a <5% cutoff was applied for all datasets, except for the data from Williams et al. 60 and from Gil-Pulido et al. 61, where <7.5% and <10% cutoffs were applied, respectively. Pre-processing code for aortic leukocyte datasets can be found as supplemental files of Zernecke et al. 34. All data were log normalized using the ‘NormalizeData’ function in Seurat with default parameters. Data integration was performed using a canonical CCA workflow in Seurat with default parameters. After CCA integration, data were scaled using ‘ScaleData’, and principal component analysis was performed using ‘RunPCA’. Dimensional reduction was performed using ‘RunUMAP’ with 30 principal components. Clustering was performed using ‘FindNeighbors’ with 30 principal components, and ‘FindClusters’ with a resolution of 0.4. Mouse scRNA-seq data given in Supplementary Figure 2 were analyzed in Seurat v4.3.0. Individual datasets were pre-

processed with quality control filtering in Seurat: cells containing >200 detected genes, and genes detected in at least 3 cells were included in the analysis using the 'CreateSeuratObject' function with 'min.features = 200' and 'min.cells=3'. Quality control filtering was further performed to remove dead/damaged cells with a high proportion of mitochondrial transcripts (>10%), and outlier cells with high UMI numbers. All data were log normalized using the 'NormalizeData' function in Seurat with default parameters. Data were pooled and batch corrected using Harmony 65 within Seurat. 2,000 highly variable genes were identified using 'FindVariableFeatures' (with selection.method = "vst"). Data were scaled using 'ScaleData' with default parameters, and principal component analysis performed using 'RunPCA' with default parameters, and batch corrected using 'RunHarmony' with default parameters. Dimensional reduction was performed using 'RunUMAP(reduction = "harmony", dims = 1:20)', and clustering was performed at a 0.4 resolution using 'FindNeighbors(reduction = "harmony", dims = 1:20)' followed by 'FindClusters(resolution = 0.2)'. Positive marker genes for each cluster were identified using 'FindAllMarkers'. Human scRNA-seq data given in Figure 7 were analyzed in Seurat v3 62 starting from the author provided cell-count matrix (downloaded from Gene Expression Omnibus GSE131778). Cells containing <200 detected genes were excluded, and genes detected in at least 3 cells were included in the analysis using the 'CreateSeuratObject' functions with 'min.features = 200' and 'min.cells=3'. Further quality control filtering was performed and cells with >5% mitochondrial transcripts were excluded, as well as cells with outlier number of UMIs (nCount_RNA>15,000). A total of 10,934 cells were analyzed. As a pre-analysis indicated a substantial patient-driven batch effect, we performed batch correction using Harmony 65 within Seurat, considering each patient as an independent sample. Data were normalized using the 'NormalizeData' function in Seurat with default parameters. 2,000 highly variable genes were identified using 'FindVariableFeatures' (with selection.method = "vst"). Data were scaled using 'ScaleData' with default parameters, and principal component analysis performed using 'RunPCA' with default parameters, and batch corrected using 'RunHarmony' with default parameters. Dimensional reduction was performed using 'RunUMAP(reduction = "harmony", dims = 1:20)', and clustering was performed at a 0.4 resolution using 'FindNeighbors(reduction = "harmony", dims = 1:20)' followed by 'FindClusters(resolution = 0.4)'. Positive marker genes for each cluster were identified using 'FindAllMarkers'. For microbiota sequencing analysis, sequences were quality filtered using the dada2 software package (version 1.12.1) 68 in the R programming language (R version 3.6.1) to produce amplicon sequence variants (ASVs). Taxonomic classification was performed using the Silva reference database (version 132) 69. Bacterial ASVs that could not be assigned to Phylum-level taxonomy were excluded. Alpha diversity was estimated using the number of observed species and the Shannon diversity index. Raw sequence data are accessible in the Sequence Read Archive (accession number pending). Beta diversity analysis was performed on proportion-normalized data using the Bray-Curtis index. Assessment for significant differences between clusters was performed using PERMANOVA with the adonis function in the vegan package (version 2.5-6) in R with 99999 permutations.
Differential abundance was tested using linear discriminant analysis with effect size (Lefse) using default settings 21.

For manuscripts utilizing custom algorithms or software that are central to the research but not yet described in published literature, software must be made available to editors and reviewers. We strongly encourage code deposition in a community repository (e.g. GitHub). See the Nature Portfolio guidelines for submitting code & software for further information.

Data

Policy information about availability of data

All manuscripts must include a data availability statement. This statement should provide the following information, where applicable:

- Accession codes, unique identifiers, or web links for publicly available datasets
- A description of any restrictions on data availability
- For clinical datasets or third party data, please ensure that the statement adheres to our policy

RNA-sequencing data of blood monocytes generated for this report has been deposited in Gene Expression Omnibus (https://www.ncbi.nlm.nih.gov/geo/query/acc.cgi?acc= GSE221782). Mouse scRNA-seq data were downloaded from Gene Expression Omnibus GSE155513 (ref 18), GSM4705592 (ref 18), GSM4705593 (ref 18), GSM4705594 (ref18), GSM4705595 (ref 18), GSM4705596 (ref 18), GSM4705597 (ref 18), GSM4705598 (ref 18), GSM4705599 (ref 18), GSE131780 (ref 33). Microbiota 16s RNA data are accessible with the following link https://www.ncbi.nlm.nih.gov/sra/PRJNA986053. Human carotid atherosclerosis scRNA-seq data were downloaded from https://figshare.com/s/c00d88b1b25ef0c5c788 (ref 71). Human coronary atherosclerosis scRNA-seq data were downloaded from Gene Expression Omnibus GSE131776 (ref 33) & GSE131778 (ref 33).

Human research participants

Policy information about studies involving human research participants and Sex and Gender in Research.

Reporting on sex and gender

For CARD-9 related pathways in human, we compared the transcriptomic profile of blood monocytes from CARD9-deficient patients (n=3, 2 males and 1 female) with those of controls (n=4, 2 males and 2 females).

Population characteristics

we compared the transcriptomic profile of blood monocytes from CARD9-deficient patients (n=3, 2 males and 1 female) with those of controls (n=4, 2 males and 2 females) . Genetic investigations in CARD9-deficient patients identified a homozygous c.865C>T in exon 6 of CARD9 leading to a premature stop codon at position 289 (p.Q289*) (N=2, 1 male and 1 female) and a homozygous c.52C>T missense mutation in exon 2 of CARD9, resulting in the replacement of the arginine in position 18 with a tryptophan residue (p.R18W) (N=1 female)

Recruitment

For human vascular tissue, patients were recruited in vascular surgery department in Paris (G Pompidou Hospital) and in Utrech (University Medical Center Utrecht)
For human monocyte studies, patients were recruited in Imagine Institute (Paris, France) in collaboration with infectious disease department (Necker Hospital, Paris, France)

Ethics oversight

Immunostaining studies were performed on arteries obtained after surgery (Ethical Committee CPP Ile de France 2013-13-19) and written informed consent was obtained. Protein of plaques were from the Athero-Express study, a longitudinal vascular biobank study in which participants provided written informed consent, and the study was approved by the Medical Ethics Committee of the University Medical Center Utrecht (NL45885.041.13, METC 13/597, Medical Ethical Committee of University). Blood monocytes were obtained from a longitudinal biobank study in which participants provided

written informed consent, and the study was approved by the ethical Committee for the Protection of Human Subjects in Biomedical Research (Inserm N° C10-14).

Note that full information on the approval of the study protocol must also be provided in the manuscript.

Field-specific reporting

Please select the one below that is the best fit for your research. If you are not sure, read the appropriate sections before making your selection.

☒ Life sciences ☐ Behavioural & social sciences ☐ Ecological, evolutionary & environmental sciences

For a reference copy of the document with all sections, see nature.com/documents/nr-reporting-summary-flat.pdf

Life sciences study design

All studies must disclose on these points even when the disclosure is negative.

Sample size	For atherosclerotic lesions characterization, sample size was determined based on the scientific litterature and our expertise. When possible, depending on the availability of the genetically-modified animals and colony production efficiency, a minimum of 10 mice were used per group to obtain statistically relevant cohorts.
Data exclusions	No data exclusion
Replication	In vivo experiments performed in mice involving different mouse models to assess the effect of Card-9 genetic deletion on atherosclerosis development were replicated 2 or 3 times depending on the model. This information is provided for each figure in the figure legends.
Randomization	No randomization
Blinding	The investigators were blinded to group allocation during data collection and analysis.

Reporting for specific materials, systems and methods

We require information from authors about some types of materials, experimental systems and methods used in many studies. Here, indicate whether each material, system or method listed is relevant to your study. If you are not sure if a list item applies to your research, read the appropriate section before selecting a response.

Materials & experimental systems

n/a	Involved in the study	
☐	☒	Antibodies
☒	☐	Eukaryotic cell lines
☒	☐	Palaeontology and archaeology
☐	☒	Animals and other organisms
☐	☒	Clinical data
☒	☐	Dual use research of concern

Methods

n/a	Involved in the study	
☒	☐	ChIP-seq
☐	☒	Flow cytometry
☒	☐	MRI-based neuroimaging

Antibodies

Antibodies used	The following antibodies provided by Cell Signaling Technology were used : phospho-AMPKa (#50081, clone D4D6D), AMPKa (#5831, clone D5A2), phospho-ACC1 (#3661), ACC1 (#3676, clone C83B10), phospho-LKB1 (#3482, clone C67A3), LKB1 (#3041, clone D60C5), phospho-Beclin-1 (#35955, clone E1C4X), Beclin-1 (#3495, clone D40C5), p62 (#23214, clone D6M5X), CHOP (#5554, clone D46F1), phospho-S6 Ribosomal Protein (#4858, clone D57.2.2E), S6 Ribosomal Protein (#2217, clone 5G10), LC3b #2775. The following antibodies provided by Abcam were used : CD36 (#ab23680, clone JC63.1), CARD9. The following antibodies provided by Novusbio were used : CHC17 (#NB120-11331, clone TD.1). The following antibodies provided by Abcam were used : CD36 (#ab23680, clone JC63.1), CARD9. The following antibodies provided by Sigma were used : beta-actin (#A5441, clone AC-15). The following antibodies provided by Dako Agilent were used : CD68, alpha-SMA. The following antibodies provided by BD Biosciences were used : FITC/ef450-coupled anti-CD11b (clone M1/70), V-500- coupled anti-B220 (#561226, clone RA63-6B2), AF-700- coupled anti-CD8a (#557959, clone 53-6.7), PerCP- coupled anti-CD45 (#557235, clone 30-F11), PE-Cy7- coupled anti-CD11c (#561022, clone HL3). The following antibodies provided by eBioscience were used : PE- coupled anti-CD115 (#12-1152-82, clone AFS98), PerCP-Cy5.5- coupled anti-Gr1 (#45-5931-80, clone RB6-8C5), PE-Cy7 coupled anti-CD4 (#25-0041-82, clone GK1.5), APC/PE-Cy7- coupled anti-NK1.1 (#25-5941-82, clone PK136), FITC/ef450- coupled anti-F4/80 (#48-4801-82, clone BM8), APC/PerCP- coupled anti-CD3ε (#17-0031-82, clone 145-2C11), FITC- coupled anti-MHC II (#11-5321-82, clone M5/114.15.2), Percp-eFluor710- coupled anti-IgM (#46-9998-42, clone SA-DA4), PE-coupled anti-CD36 (#12-0362-82, clone HM36).
Validation	The primary antibodies have been used according to the manufacturer's website instructions.

Animals and other research organisms

Policy information about studies involving animals; ARRIVE guidelines recommended for reporting animal research, and Sex and Gender in Research

Laboratory animals	C57BL/6 Card9-/-, Card9-/-;Rag2-/-, Apoe-/-;Card9-/-, Apoe-/-;Card9-/-;Rag2-/-, CD36-/-;Card9-/- male mice were used for the characterization of atherosclerotic lesions and were included in the experiment at the age of 6 weeks. 10 weeks-old C57BL/6 LDLr-/- males and females were used for the bone marrow transplantation experiments.
Wild animals	NA
Reporting on sex	C57BL/6 Card9-/-, Card9-/-;Rag2-/-, Apoe-/-;Card9-/-, Apoe-/-;Card9-/-;Rag2-/-, CD36-/-;Card9-/- male mice were used for the characterization of atherosclerotic lesions and were included in the experiment at the age of 6 weeks. 10 weeks-old C57BL/6 LDLr-/- females were used for the Card9+/+ vs Card9-/- bone marrow transplantation experiments. 10 weeks-old C57BL/6 LDLr-/- males were used for the CD36-/-Card9+/+ vs CD36-/-Card9-/- bone marrow transplantation experiments.
Field-collected samples	NA
Ethics oversight	Experiments were conducted according to the guidelines formulated by the European Community for experimental animal use (L358-86/609EEC) and were approved by the Ethical Committee of INSERM and the French Ministry of Agriculture (agreement A75-15-32).

Note that full information on the approval of the study protocol must also be provided in the manuscript.

Clinical data

Policy information about clinical studies

All manuscripts should comply with the ICMJE guidelines for publication of clinical research and a completed CONSORT checklist must be included with all submissions.

Clinical trial registration	NA
Study protocol	NA
Data collection	NA
Outcomes	NA

Flow Cytometry

Plots

Confirm that:

☒ The axis labels state the marker and fluorochrome used (e.g. CD4-FITC).

☒ The axis scales are clearly visible. Include numbers along axes only for bottom left plot of group (a 'group' is an analysis of identical markers).

☒ All plots are contour plots with outliers or pseudocolor plots.

☒ A numerical value for number of cells or percentage (with statistics) is provided.

Methodology

Sample preparation	Blood and spleen samples were collected at sacrifice for analysis of leukocyte subsets.
Instrument	Cells were acquired using a BD LSRII Fortessa flow cytometer (BD Biosciences)
Software	Cells were analyzed with FlowJo™ (TreeStar, Inc.).
Cell population abundance	Minimum of 0.5 million of cells were analyzed in each flow cytometry sample
Gating strategy	Myeloid cells were identified as CD45+CD11b+. Monocytes were identified as CD11b+CD115+. Among them, classical monocytes were Gr1high (or Ly6Chigh) and non-classical monocytes were Gr1low (or Ly6Clow). Neutrophils were identified as CD11b+CD115-Gr1+ (or CD11b+CD115-Ly6G+). B220+IgM+ B lymphocytes, CD4+ and CD8+ T lymphocyte subsets were also analyzed. Antibodies raised against CD11b, CD115, Gr1 (Ly6C and G), B220, CD4, CD8a, NK1.1, CD45, F4/80, CD3ε, MHC II, IgM, CD11c and CD36 were used for immunostaining.

☒ Tick this box to confirm that a figure exemplifying the gating strategy is provided in the Supplementary Information.
